# Use of synthetic biology tools to optimize the production of active nitrogenase Fe protein in chloroplasts of tobacco leaf cells

**DOI:** 10.1111/pbi.13347

**Published:** 2020-04-07

**Authors:** Álvaro Eseverri, Gema López‐Torrejón, Xi Jiang, Stefan Burén, Luis M. Rubio, Elena Caro

**Affiliations:** ^1^ Centre for Plant Biotechnology and Genomics Instituto Nacional de Investigación y Tecnología Agraria y Alimentaria (INIA) Universidad Politécnica de Madrid (UPM) Madrid Spain; ^2^ Departamento de Biotecnología-Biología Ve ge tal Escuela Técnica Superior de Ingeniería Agronómica Alimentaría y de Biosistemas Universidad Politécnica de Madrid Madrid Spain

**Keywords:** synthetic biology, nitrogenase, Fe protein

## Abstract

The generation of nitrogen fixing crops is considered a challenge that could lead to a new agricultural ‘green’ revolution. Here, we report the use of synthetic biology tools to achieve and optimize the production of active nitrogenase Fe protein (NifH) in the chloroplasts of tobacco plants. *Azotobacter vinelandii* nitrogen fixation genes, *nifH*, *M*, *U* and *S,* were re‐designed for protein accumulation in tobacco cells. Targeting to the chloroplast was optimized by screening and identifying minimal length transit peptides performing properly for each specific Nif protein. Putative peptidyl‐prolyl cis‐trans isomerase NifM proved necessary for NifH solubility in the stroma. Purified NifU, a protein involved in the biogenesis of NifH [4Fe‐4S] cluster, was found functional in NifH reconstitution assays. Importantly, NifH purified from tobacco chloroplasts was active in the reduction of acetylene to ethylene, with the requirement of *n* *ifU* and *n* *ifS* co‐expression. These results support the suitability of chloroplasts to host functional nitrogenase proteins, paving the way for future studies in the engineering of nitrogen fixation in higher plant plastids and describing an optimization pipeline that could also be used in other organisms and in the engineering of new metabolic pathways in plastids.

## Introduction

Nitrogen is a critically important element for all organisms, the element that is required in amounts greater than all others except for carbon, oxygen and hydrogen. It is very abundant in the atmosphere, which is composed of a 78% of N_2_ gas; however, neither plants nor animals can obtain nitrogen directly from N_2_. Instead, they ultimately depend on a process performed by some archaea and bacteria known as biological nitrogen fixation, where N_2_ is converted into the usable form of ammonia (NH_3_) by the nitrogenase enzyme.

Nitrogen availability often limits the productivity of natural and managed ecosystems, so much so, that among all farmer‐controlled input factors, nitrogen is considered to have the second‐largest impact on plant growth, only after water (Martin and Marschner, 2006). To increase crop yield, the use of nitrogen fertilizers has become widespread in developed countries, with enormous undesired environmental consequences that cannot be overlooked. On the other hand, the cost of chemical fertilizers is prohibitive for farmers in the developing countries, which is why there are regions in Sub‐Saharan Africa and Southeast Asia where crop yields are extremely low and poverty and hunger prevail (Mueller *et al.*, 2012).

The use of biotechnology to boost biological nitrogen fixation has been considered, for at least the last 45 years, as one of the best strategies to increase crop yield to face population growth and general agronomic crop demand while preserving the environment (Beatty and Good, 2011; Burén and Rubio, 2018; Curatti and Rubio, 2014; Hardy and Havelka, 1975; Ladha and Reddy, 1995; Oldroyd and Dixon, 2014; Rogers and Oldroyd, 2014; Rosenblueth *et al.*, 2018). In this context, one of the proposed biotechnological strategies involves the direct transfer of the prokaryotic genes needed to assemble a functional molybdenum nitrogenase (*nif* genes) into the plant genome, a huge undertaking given the relative large number of genes in nitrogenase biosynthetic pathway and the extreme O_2_ sensitivity of its components and the proteins involved in the synthesis of their metal cofactors (Curatti and Rubio, 2014). The molybdenum nitrogenase is composed of an Fe protein (encoded by *nifH*) and a MoFe protein (encoded by *nifD* and *nifK*), which require iron–sulphur clusters for activity. Since the nitrogenase Fe protein is the most sensitive component to oxygen, and its maturation requirements are simpler and better characterized than those of the MoFe protein, it has been chosen as a proof of concept by most of the previous efforts to express *nif* genes in eukaryotes. There are four genes in *Azotobacter vinelandii* required to render active Fe protein (*nifH, nifM, nifU* and *nifS*) (Jacobson *et al.*, 1989). NifM is a putative peptidyl‐prolyl cis‐trans isomerase that assists in the proper folding of NifH (Gavini *et al.*, 2006). NifS is a cysteine desulfurase responsible for the mobilization and transfer of sulphur to NifU (Yuvaniyama *et al.*, 2000; Zheng *et al.*, 1993), where [Fe‐S] clusters are synthesized and later donated to the Fe protein for activation (Dos Santos *et al.*, 2004). This is an oxygen‐sensitive process in which intermediates are generated and carried directly from one protein to another to protect them from reactive species (Zheng and Dos Santos, 2018).

The most successful effort in transferring active nitrogenase components to eukaryotes to date was the production of active NifU and NifH within the mitochondria of yeast cultures growing aerobically, overcoming the O_2_ sensitivity of Nif proteins (López‐Torrejón *et al.*, 2016). After that, Allen *et al. *(2017) showed that indeed, 16 *nif‐* or *nif*‐related genes from *Klebsiella oxytoca* could be individually expressed fused tomitochondrial targeting peptides to accumulate in the mitochondrial matrix of tobacco cells. However, no protein activity was demonstrated.

A different location where to engineer nitrogen fixation in plants is chloroplast, suggested already in 1984 by Merrick and Dixon because of their availability of the immediate products of photosynthesis, NADPH and ATP, that could satisfy nitrogen fixation demands of energy and reducing power. Moreover, the ammonia produced as a consequence of nitrogen fixation could be easily assimilated into the formation of amino acids by the glutamine synthetase/glutamate synthase pathway, found in plastids. In 1997, Dixon et al. expressed *nifH* and *nifM* in tobacco leaf chloroplasts. Even though it was shown that the proteins were stable when targeted to this organelle by transit peptides, they accumulated at levels too low for functionality tests. Using a different strategy, Cheng *et al. *(2005) transformed directly the chloroplast genome and showed that NifH was expressed and accumulated in the chloroplasts of the unicellular alga *Chlamydomonas reinhardtii*. NifH was not fully functional but, although Fe protein activity was not proven, it could partially substitute for the function of the putative ‘chlorophyll iron protein’ encoded by *chlL,* a protein similar to NifH in both structure and function and also sensitive to oxygen. More recently, Ivleva *et al. *(2016) reported the generation of transplastomic tobacco plants expressing *nifH* and *nifM*. Their approach rendered a high amount of protein accumulating in the leaves of the plants, but NifH activity could only be detected when plants were incubated under low oxygen conditions (10% O_2_), and even then, the activities obtained were very low.

Thus, although it has been possible to express some nitrogenase proteins in chloroplasts, there are still problems to overcome. While direct transformation of the chloroplast would be ideal (Adem *et al.*, 2017), since chloroplast transformation methods are not yet in place for maize and very few successful applications have been developed for rice, we have focused our work on the expression of nuclear‐encoded *nif* genes. In this context, increasing *nif* gene expression and optimizing import of the recombinant proteins into chloroplasts were our main priorities in the pursuit of active Fe protein in chloroplasts, using a strategy that can be directly transferred to cereals.

It is well known that the DNA sequence used to encode a protein can have dramatic effects on its expression and accumulation (Gustafsson *et al.*, 2012). mRNA structures near the ribosome binding site were long ago reported to reduce translational initiation (Kozak, 1986), as well as mRNA destabilization by the basal mRNA decay machinery and sequence‐specific decay components (Gutiérrez *et al.,* 1999). The exclusion of these elements from synthetic genes, together with premature polyadenylation sites and cryptic intron splice sites, has been described to increase transcription and enhance translation in plants (Jackson *et al.*, 2014). In an attempt to design synthetic *nif* genes with a higher expression and stability, we eliminated these undesired elements from their sequence and had them match codon usage and GC content from the host as much as possible, which is well known to affect recombinant protein expression (Webster *et al.*, 2017).

Another step that could be limiting the availability of Nif proteins in the chloroplast is their import from the cytosol. It requires chloroplast targeting peptides (CTPs), N‐terminal targeting sequences that direct proteins to their correct subcellular compartment and, after import through an ATP hydrolysis‐dependent process, are cleaved by the stromal processing peptidase. The targeting peptide of the small subunit of RuBisCo, the most commonly used CTP, is very efficient driving most recombinant proteins to the chloroplast stroma, although the N‐terminal region of cargo proteins is also critical for CTP activity (Shen *et al.*, 2017).

In the work presented here, the generation of synthetically designed genes to maximize Nif protein accumulation and the screening for the optimal chloroplast transit peptide for each Nif protein, led to tobacco plants where NifH, M, U and S accumulated as soluble proteins within the stroma of mesophyll cells and protein activity could be shown for NifU and NifH.

## Results

### *nif* gene synthetic design

Previous studies had shown that nuclear‐encoded *n* *if* *H* could not be expressed to high levels in tobacco chloroplasts using its original sequence from *K. oxytoca* (Dixon *et al.*, 1997). The codon adaptation index (CAI) is a simple and effective way of measuring synonymous codon usage bias and the likeliness of success of heterologous gene expression (Sharp and Li, 1987). The sequences of *nifH, nifM, nifU* and *nifS* codon optimized for their expression in *Saccharomyces cerevisiae* (López‐Torrejón *et al.*, 2016) showed a theoretical good optimization also for expression in tobacco, with a CAI of 0.82, 0.84, 0.81 and 0.82, respectively, and no presence of low frequency (<40%) codons (Biologics International Corp codon adaptation index calculator and rare codon analyser). These values strongly indicated that heterologous gene expression in tobacco using the yeast codon‐optimized sequences could be successful, especially when compared with the data for the original Azotobacter genes (CAIs of 0.60, 0.54, 0.55 and 0.53 and presence of 5, 16, 10 and 12 low frequency codons for *nifH, nifM, nifU* and *nifS,* respectively).

In addition, we decided to design *nif* synthetic genes specifically optimized for maximum expression in tobacco, taking into account, not only codon usage patterns, but also other additional parameters, such as mRNA stability motifs, hidden stop codons and poly(A) signals that could impact protein production. The web server application Codon Optimization OnLine (COOL) (Chin *et al.*, 2014) was used for this purpose.

The specific parameters of the synthetic design used in this work involved using an individual codon usage and a GC content matching that of tobacco coding sequences, both in general and specifically in the third base of codons (GC3) (Nakamura *et al.*, 2000). Moreover, some motifs were actively avoided, like any form of dicotyledonous plant polyadenylation signal, reported RNA instability motifs and repetitions of more than 7 bp or A and T strings (Jackson *et al.*, 2014; Ji *et al.*, 2007; Shen *et al.*, 2008) (Table S1).

**Figure 1:**
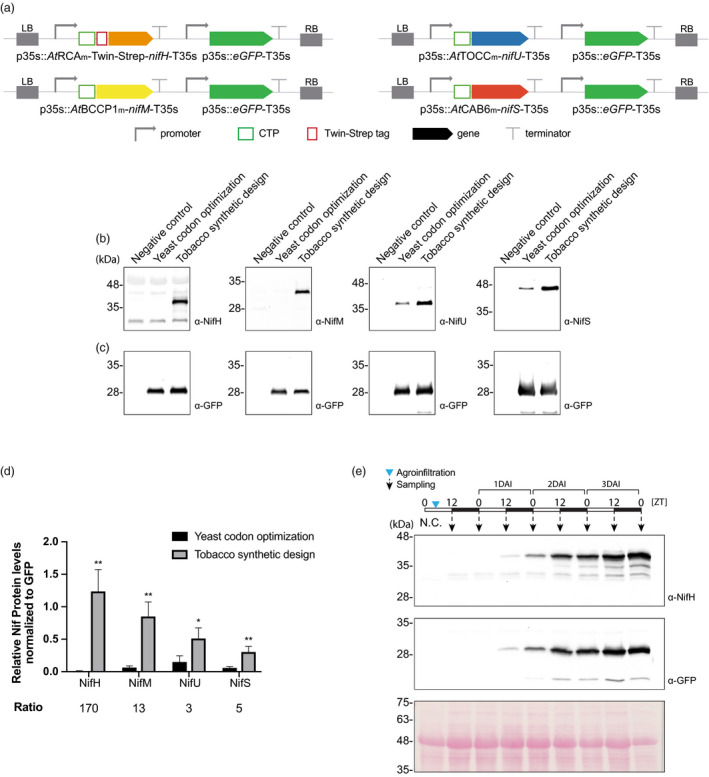
Effect of using synthetically designed *n* *if* genes optimized for tobacco. (a) Schematic representation of the constructs used in *N. benthamiana*‐transient expression assays leading to *nif* genes and GFP simultaneous expression. (b) Western blot analysis of NifH, NifM, NifU and NifS accumulation when expressed from yeast codon‐optimized genes or synthetically designed genes optimized for expression in tobacco. Negative control refers to *N. benthamiana* leaves agroinfiltrated with Agrobacterium transformed with an empty vector. Expected sizes are Twin‐Strep NifH: 35.3 kDa, NifM: 32.3 kDa, NifU: 34.4 KDa and NifS: 44.3 kDa. (c) Western blot analysis of GFP accumulation. Expected size: 27kDa. (d) Quantification of Nif/GFP accumulated protein. Mean ± SD (*n* = 3 biological replicates). ‘Ratio’ refers to the ratio between Nif protein levels achieved using the tobacco synthetic design and the yeast codon‐optimized sequences.* Represents Student’s test significant differences (*P* < 0.05); ** represents Student’s test highly significant differences (*P* < 0.01). (e) Time course of NifH (tobacco synthetic design) and GFP accumulation after *N. benthamiana* agroinfiltration. N.C. refers to ‘negative control’, *N. benthamiana* leaves agroinfiltrated with Agrobacterium transformed with an empty vector.

We generated multigenic constructs comprised by a transcriptional unit where the p35S promoter directs the expression of each yeast codon‐optimized or synthetically designed *nif* gene and a second transcriptional unit where the p35S directs the expression of GFP. Transient expression assays were performed to gauge the effect of the synthetic gene design (Figure 1a). The accumulation of Nif proteins (Figure 1b) was compared, using the level of GFP to normalize for differences in overall recombinant protein expression (Figure 1c). The results showed increased accumulation of all four Nif proteins when using the constructs with the synthetically optimized genes, validating the parameters established for their re‐design (Figure 1d). The increase was specifically significant in the case of NifH that accumulated 170 times more when the synthetic gene was used for expression (Figure 1d).

We followed the accumulation of NifH through a time course from the moment of agroinfiltration to optimize the time of tissue collection, and found that NifH levels steadily increase until the end of the dark period of the third day (Figure 1e). This is in accordance with the standard method for transient expression in *N. benthamiana* (Li, 2011; Naim *et al.*, 2012) and shows that the oxygen derived from the photosynthesis taking place during the light period of plant growth did not lead to a decrease in NifH accumulation.

### Optimization of chloroplast targeting

In an effort to find the CTP that best suited each specific Nif protein in study, twelve different CTPs previously used in the literature to drive protein import into the chloroplast stroma were fused to each Nif protein, namely *At*AROA_CTP_ (Klee *et al.*, 1987), *At*BCCP1_CTP_, *At*CAB6_CTP_, *At*DNAJ8 _CTP_, *At*GLTB2_CTP_, AtTOCC_CTP _(Lee *et al.*, 2008), *Nt*RBS_CTP_(Mazur and Chui, 1985), *At*RBS1A_CTP_(Lee and Hwang, 2011), *Ps*RBS2_CTP_(Coruzzi *et al.*, 1984), *At*RCA_CTP_ (Kim *et al.*, 2010), *Nt*SIR_CTP _ (Yonekura‐Sakakibara *et al.*, 1998), and *syn*RBS_CTP_ (Engler *et al.*, 2014) (details in Table S2) and analysed for protein accumulation upon agroinfiltration of *N. benthamiana* leaves.

**Figure 2:**
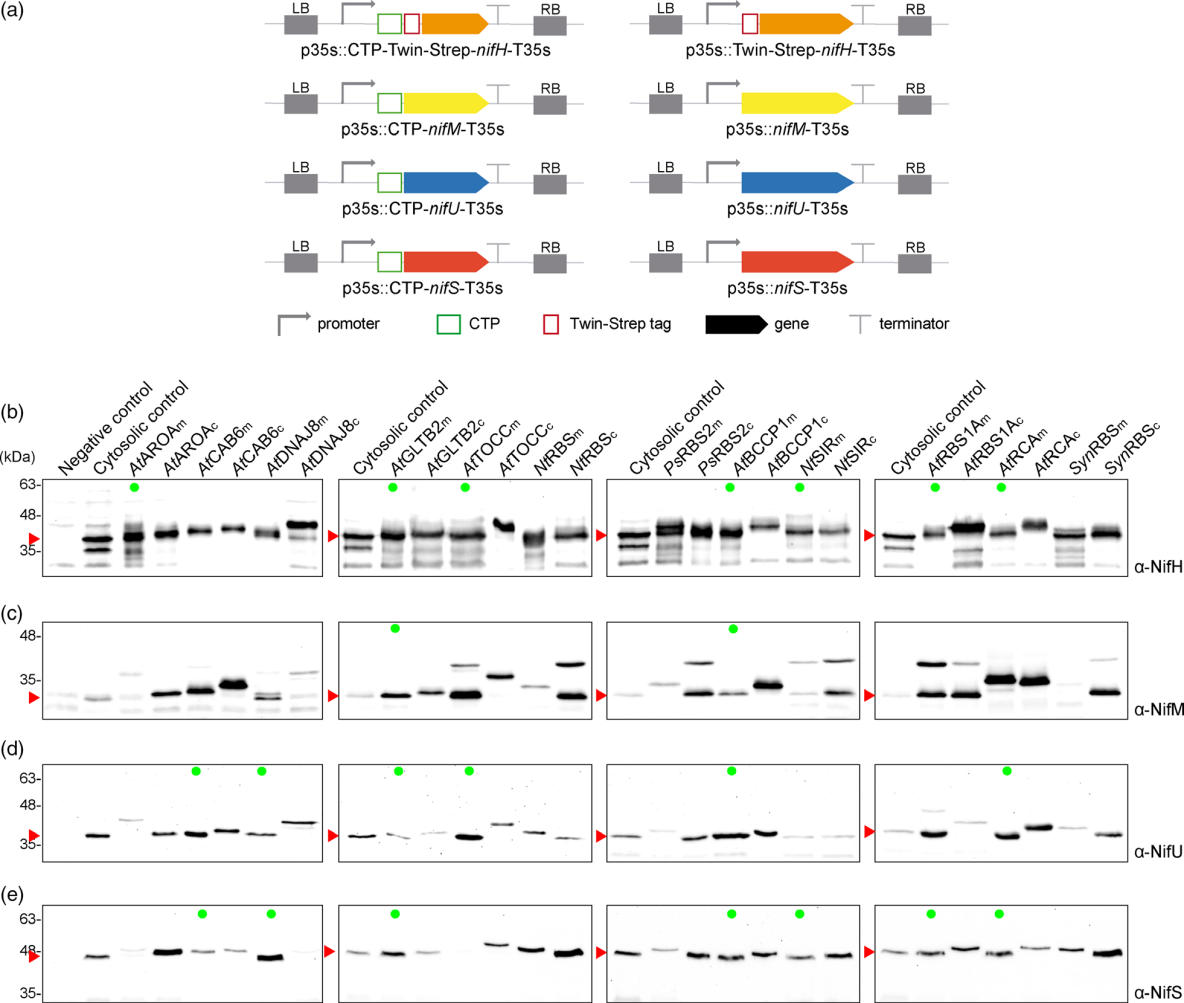
Screening of chloroplast targeting peptides fused to NifH, NifM, NifU and NifS proteins. (a) Schematic representation of the constructs used in *N. benthamiana*‐transient expression assays leading to CTP‐*nif* expression. (b–e) Western blot analysis of the product of expressing 12 CTPs in full version (complete) and up until their predicted cleavage site by stromal peptidase (minimal) fused to the N‐terminus of NifH (b), NifM (c), NifU (d) and NifS (e). Negative control refers to *N. benthamiana* leaves agroinfiltrated with Agrobacterium transformed with an empty vector. Cytosolic versions were expressed for Nif correct size control, expected sizes are Twin‐Strep NifH: 35.3 kDa, NifM: 32.3 kDa, NifU: 34.4 KDa and NifS: 44.3 kDa. For CTP sizes, see Table S2. Green dots mark Nif proteins with the same electrophoretic mobility as their cytosolic controls, consistent with complete cleavage of the CTP. The triangles in red mark the expected mature form of the protein.

The use of CTP sequences that extend beyond the peptidase cleavage site (complete CTP, c) would produce recombinant proteins with additional amino acids left at the N‐terminus after CTP cleavage. Since this can affect protein functionality, shorter CTP versions were generated by cloning only the CTP coding sequence up until the cleavage recognition site predicted by ChloroP 1.1 server (Emanuelsson *et al.*, 1999) (minimal CTP, m). Constructs for expression of cytosolic Nif proteins (protein size control) and Nif fusions to complete and minimal versions for all twelve candidate CTPs were generated (Figure 2a).

Obtaining Nif proteins with the same electrophoretic mobility as the cytosolic versions (Figure 2b–e, green dots) suggested correct import and cleavage of the CTP, generating a chloroplast‐recombinant Nif protein with none, or very few, residual amino acids in the N‐terminus. Nif proteins with an increased size coinciding with that of the Nif protein plus the CTP suggested neither import nor cleavage had taken place. The appearance of two bands could be indicative of partial import and cleavage (Figure 2b–e).

**Figure 3:**
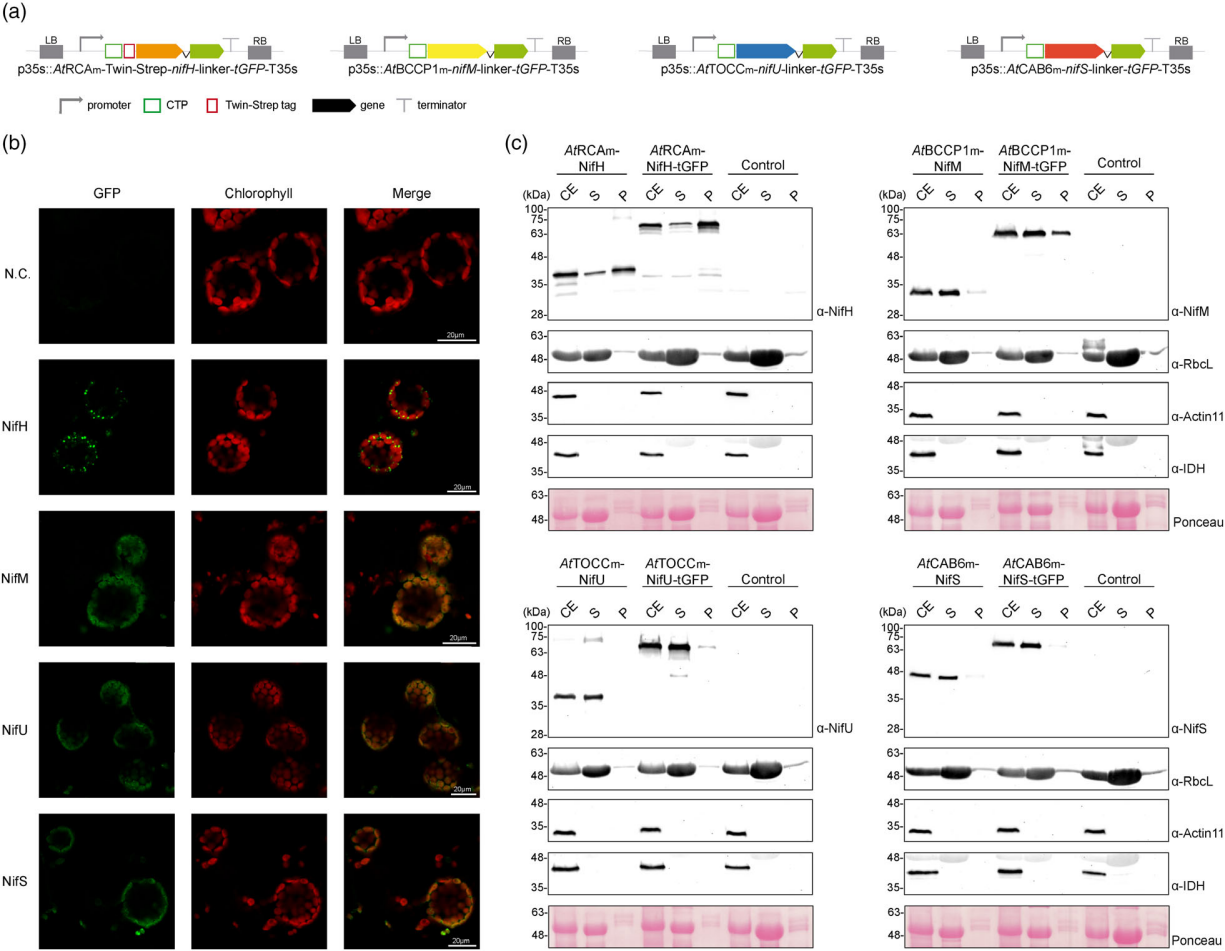
Localization of stroma‐targeted Nif proteins. (a) Schematic representation of the constructs used in *N. benthamiana*‐transient expression assays leading to Nif localization. (b) Confocal microscopy images of Nif proteins fused to tGFP. N.C. corresponds to ‘negative control’, a non‐agroinfiltrated leaf. The three columns show the individual signals for tGFP (green, on the left) and chlorophyll autofluorescence (red, in the centre). On the right, overlap of both signals (merge). (c) Chloroplast purification assays. Total proteins extract prior to chloroplast purification (CE). Intact chloroplasts were isolated and then broken to separate the soluble (S) and membrane‐associated (P) fractions. Control refers to *N. benthamiana* plants agroinfiltrated with agrobacterium transformed with an empty vector. Proteins were resolved by SDS‐PAGE and detected by Western blot. Expected sizes are Twin‐Strep NifH: 35.3 kDa, NifM: 32.3 kDa, NifU: 34.4 kDa, NifS: 44.3 kDa (27 kDa more in the case of tGFP fusions), RbcL: 52.7 kDa, Actin‐11: 41.6 kDa and IDH: 39 kDa. Note that the IDH blot was re‐probed after striping anti‐RuBisco and some signal remains (upper band).

One of the best performing CTPs for each specific Nif protein was selected (*At*RCAm for NifH, *At*BCCP1m for NifM, *At*TOCCm for NifU and *At*CAB6m for NifS), and Nif proteins were fused in their C‐terminus to turbo GFP, in order to visualize them within tobacco leaf mesophyll cells (Figure 3a). NifM, NifU and NifS all showed a diffuse pattern co‐localized with chlorophyll, supporting correct targeting to the chloroplast stroma (Figure 3b). However, for NifH, we could observe GFP concentrated in foci located within or around the chloroplasts, and that excluded chlorophyll. Chloroplast purification assays were performed in leaf tissue expressing Nif or Nif‐GFP fusion proteins. NifM, NifU and NifS, as well as their corresponding GFP fusions, were confirmed to accumulate mostly in the stroma soluble fraction, while the majority of NifH and NifH‐GFP appeared associated with membranes (Figure 3c). The similar behaviour of Nif and Nif‐GFP fusions validated the results obtained by microscopy, confirming that the fusion of the reporter protein does not lead to an artifactual localization and a clear indication of a NifH solubility problem. Anti‐isocitrate dehydrogenase (IDH) antibody was used to check for the presence of mitochondrial contamination in the isolation of chloroplasts, and the negative results obtained suggest that the NifH‐GFP foci observed do not correspond to mitochondria, but rather, to chloroplast‐associated bodies.

**Figure 4:**
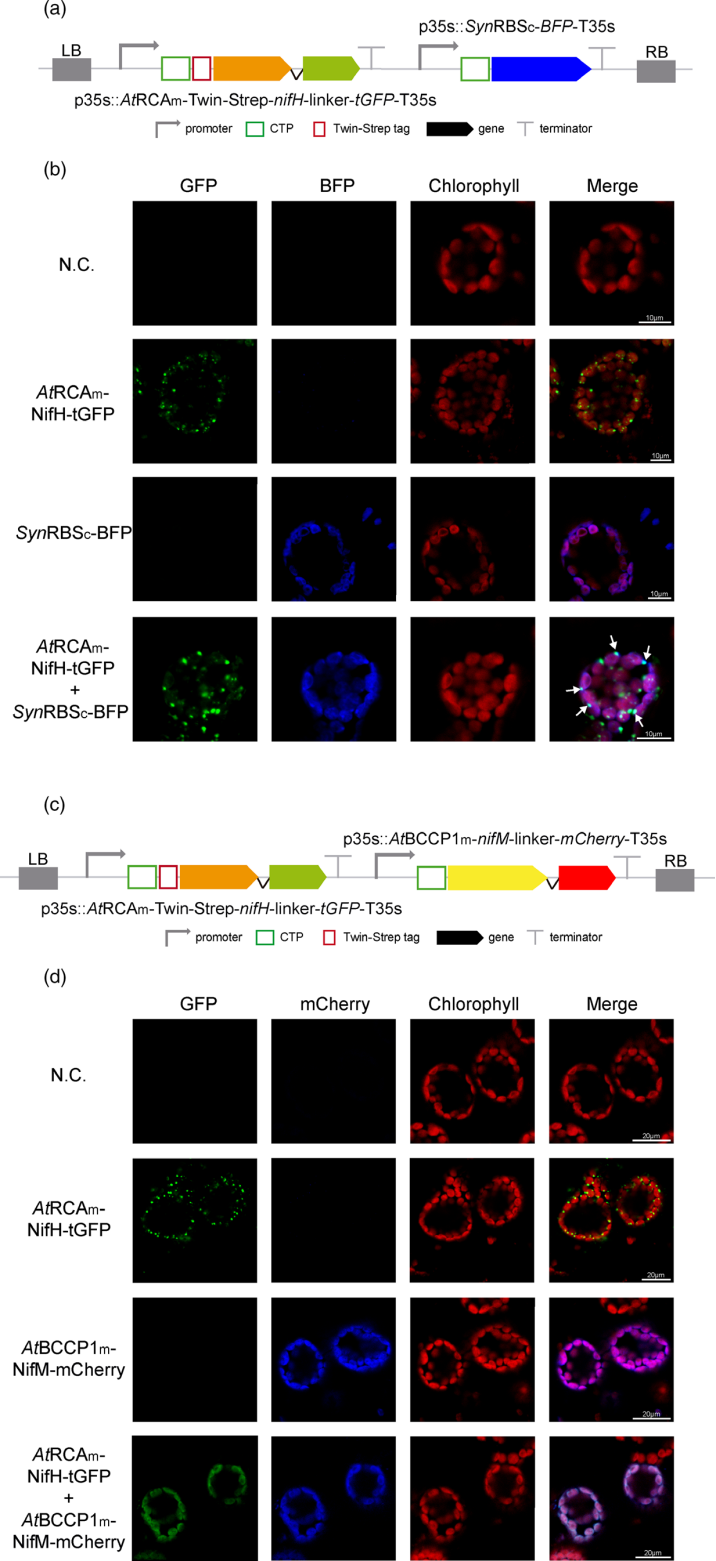
NifM is necessary for the accumulation of soluble NifH in the stroma. (a) Schematic representation of the constructs used in *N. benthamiana*‐transient expression assays leading to NifH and stromal BFP co‐localization. (b) Confocal microscopy images of NifH fused to tGFP and stromata‐targeted BFP. N.C. corresponds to ‘negative control’, a non‐agroinfiltrated leaf. The four columns show the individual signals for tGFP (green), BFP (blue) and chlorophyll autofluorescence (red). On the right, overlap of the three signals (merge). Note arrows pointing to co‐localization of green and blue signals in a punctuate pattern. (c) Schematic representation of the construct used in *N. benthamiana*‐transient expression assays leading to NifH and NifM co‐localization. (d) Confocal microscopy images of co‐expressed NifH and NifM proteins fused to tGFP and mCherry, respectively. N.C. corresponds to ‘negative control’, a non‐agroinfiltrated leaf. The four columns show the individual signals for tGFP (green), mCherry (blue) and chlorophyll autofluorescence (red). On the right, overlap of the three signals (merge). Note the co‐localization of green and blue signals in a diffuse pattern within the chloroplast.

To elucidate the nature and origin of these NifH‐accumulating foci, we co‐expressed the *At*RCAm‐NifH‐GFP fusion with a *syn*RbsSc‐BFP, that accumulates in the stroma (Figure 4a). The co‐localization of GFP and BFP in a punctuate pattern (Figure 4b, arrows) supports that after NifH is imported into the chloroplast, it is included in vesicles together with other stroma soluble proteins, like BFP.

**Figure 5:**
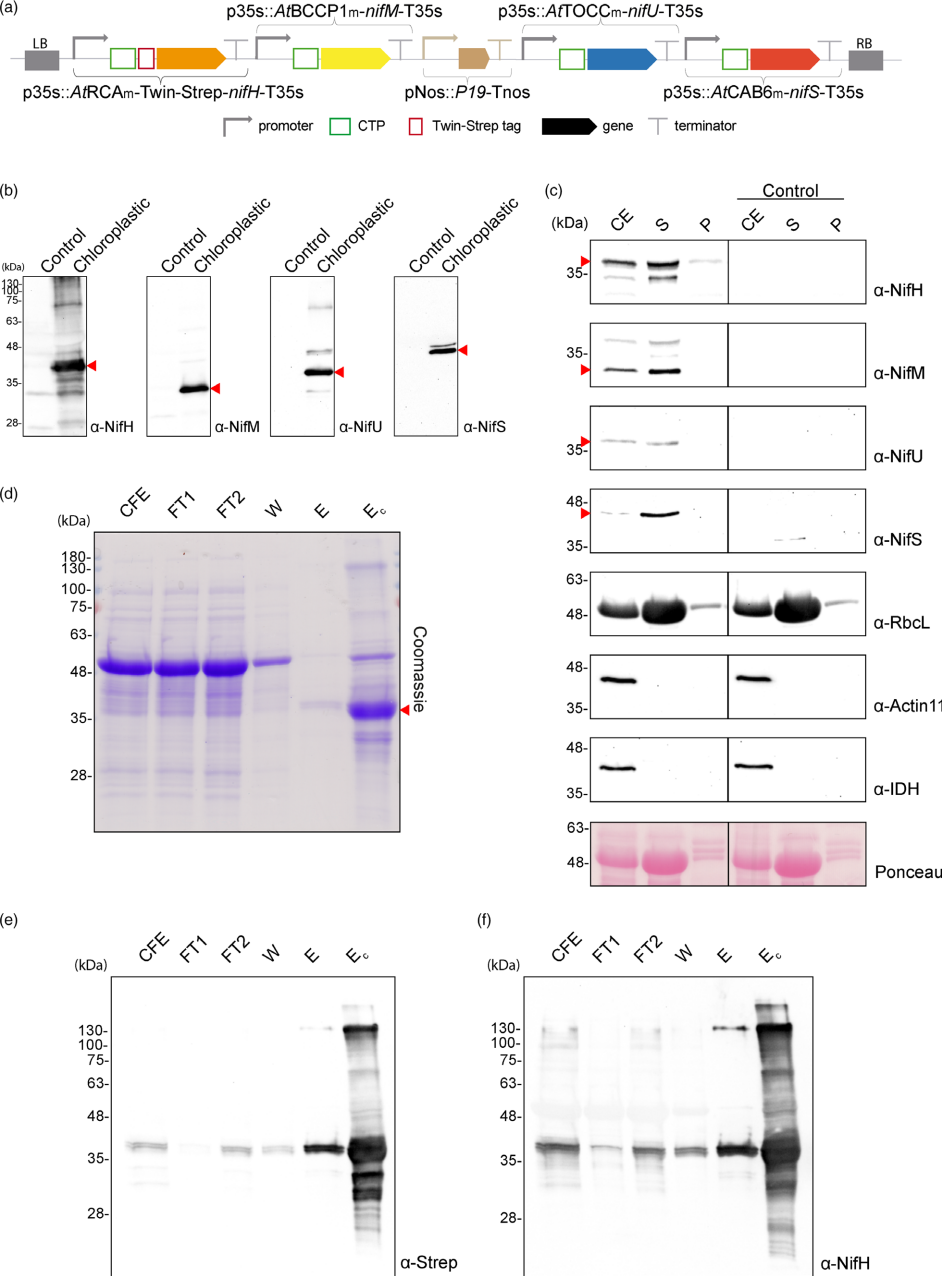
Purification of NifH from chloroplasts of *N. benthamiana* co‐expressing *nifH*, *nifM*, *nifU* and *nifS.* (a) Schematic representation of the multigenic construct used in *N. benthamiana*‐transient expression assays leading to NifH purification. (b) Western blot analysis of the agroinfiltrated tissue using antibodies targeting Strep‐tagged NifH, NifM, NifU and NifS as indicated. Expected sizes are Twin‐Strep NifH: 35.3 kDa, NifM: 32.3 kDa, NifU: 34.4 kDa and NifS: 44.3 kDa. (c) Chloroplast purification assays. Total proteins extract prior to chloroplast purification (CE). Intact chloroplasts were isolated and then broken to separate the soluble (S) and membrane‐associated (P) fractions. Control refers to *N. benthamiana* plants agroinfiltrated with agrobacterium transformed with an empty vector. Proteins were resolved by SDS‐PAGE and detected by Western blot. Expected sizes are RbcL: 52.7 kDa; Actin‐11: 41.6 kDa and IDH: 39 kDa. The triangles in red mark the mature form of the protein. (d) Coomassie staining illustrating the Strep‐Tactin purification procedure of *A. vinelandii* NifH expressed in leaves of *N. benthamiana*. CFE, cell‐free extract (soluble fraction following centrifugation and filtering of disrupted leaf tissue); FT, Strep‐Tactin flow‐through fraction; W, wash fraction; E, biotin‐eluted fraction; Ec, concentrated eluate. (e–f) Western blot analysis of the Strep‐Tactin purification procedure. Antibodies targeting Strep‐tag (e) or NifH (f) were used for visualization of the purified NifH protein.

When both *nifH* and *nifM* genes were co‐expressed (Figure 4c), NifH punctuated accumulation disappeared and could only be found diffuse within the chloroplast stroma (Figure 4d). This observation was corroborated by chloroplast purifications from cells co‐expressing *nifH*, *nifM, nifU* and *nifS*, where NifH could now be found mostly in the soluble protein fraction coming from the stroma (Figure 5a, c). All these data suggest that when expressed alone, NifH is accumulating in aggregates of immature protein and becomes included in vesicles, and that NifM is required for NifH to fold correctly and become soluble in the stroma.

### Tobacco plants co‐expressing *nifH, nifM, nifU* and *nifS* exhibit Fe protein activity

Once optimal conditions had been established for Nif protein accumulation and import to the chloroplast stroma, a multigenic construct was assembled with the tobacco synthetic *nif* genes and the selected CTPs (Figure 5a). For protein purification, a single vector containing *nifH, nifM, nifU* and *nifS* in addition to the silencing suppressor was used. Following this approach in transient expression assays assures that all cells infected by Agrobacterium will get the complete gene set, and the purified Nif protein will have been expressed and maturated in cells containing simultaneously all of them.

Tobacco leaves were collected at the end of the dark period of the third day after agroinfiltration, when all four *nif* genes were expressed (Figure 5b) and Nif proteins accumulated in soluble form within the chloroplast stroma (Figure 5c).

NifH from *N. benthamiana* chloroplasts was purified using Twin‐Strep‐tag for affinity chromatography (Figure 5d–f). Various bands could be detected in the elution fraction after NifH purification. Peptide mass fingerprinting identified them as a RuBisCo subunit (probably a contaminant), the maturase NifM and NifH degradation products or multimers (Figure S1). NifH migrated as a close double band in SDS‐PAGE (Figure 5e–f); however, it could not be due to N‐terminal degradation or unspecific cleavage of the CTP by the stromal peptidase, since Edman degradation of the purified protein confirmed a unique N‐terminal sequence starting at the predicted location for CTP cleavage (Figure S2). NifH doublets not related to post‐translational modifications have been previously observed both in diazotrophic bacteria and in heterologous hosts (Howard *et al.*, 1986; Roberts *et al.*, 1978). Detection after Western blots using anti‐NifM showed a band that corroborated some degree of co‐elution due to interaction with NifH. The same was observed using an anti‐NifU antibody (Figure S3), suggesting the on‐going maturation of a subpopulation of Fe protein in the samples.

Pure NifH exhibited a light yellowish/brownish colour, characteristic of Fe‐S proteins. Its activity was determined by using *in vitro* acetylene reduction assays in which the *N. benthamiana* NifH was incubated with MoFe protein purified from *A. vinelandii*. *N. benthamiana* Fe protein activity varied among preparations, with a maximum of specific activity of 189 nmol of ethylene formed per minute and milligram of MoFe protein at a *N. benthamiana* Fe protein/*A. vinelandii* MoFe protein molar ratio of 25 (Table 1).

**Table 1:** Maximum nitrogenase MoFe protein activity supported by *N. benthamiana* NifH and *A. vinelandii* NifH (highest result obtained in three positive independent biological experiments)

Source of Fe protein	Acetylene reduction assay†	Fe protein to MoFe protein molar ratio
*N. benthamiana* NifH	189 ± 5	25
*A. vinenlandii* NifH	1194 ± 65	25

^†^ Defined as nmol of C_2_H_4_ formed per minute and milligram of MoFe protein (mean ± SD, *n* = 2 technical replicates).

Although the activities detected are low compared to those of *A. vinelandii* NifH (a maximum of 16% activity was obtained in chloroplast NifH compared to *A. vinelandii* NifH), these results indisputably show that tobacco chloroplasts are capable of accumulating functional Fe protein, at least when its maturation and [Fe‐S] cluster assembly is separated in time from the process of photosynthesis (activity shown for samples collected at the end of the dark period). Future efforts will be directed at designing strategies to protect NifH [Fe‐S] clusters from oxygen as a means to increase its activity, and to test the effect of photosynthesis during the light period on the protein metal content and activity. The influence of the Twin‐Strep‐tag cannot be considered as a factor accounting for the low chloroplast NifH activity, since *A. vinelandii* Strep‐tagged NifH showed almost the same activity as the non‐tagged version when purified from a Δ*nifH* strain (Figure S4).

In the chloroplast, other pathways exist for Fe–S protein assembly, like the sulphur mobilization pathway (SUF), raising the question of the requirement for NifU and NifS. To test their role in the assembly and delivery of iron–sulphur clusters to NifH within the chloroplast, we performed the same experiments in *N. benthamiana* leaves expressing only *nifH* and *nifM*. In the absence of NifU and NifS, no NifH activity could be detected, suggesting that the chloroplast assembly and transfer factors are not able to provide enough [Fe‐S] clusters to result in a functional NifH (Figure S5).

### Purification of active NifU from *N. benthamiana* chloroplasts

**Figure 6:**
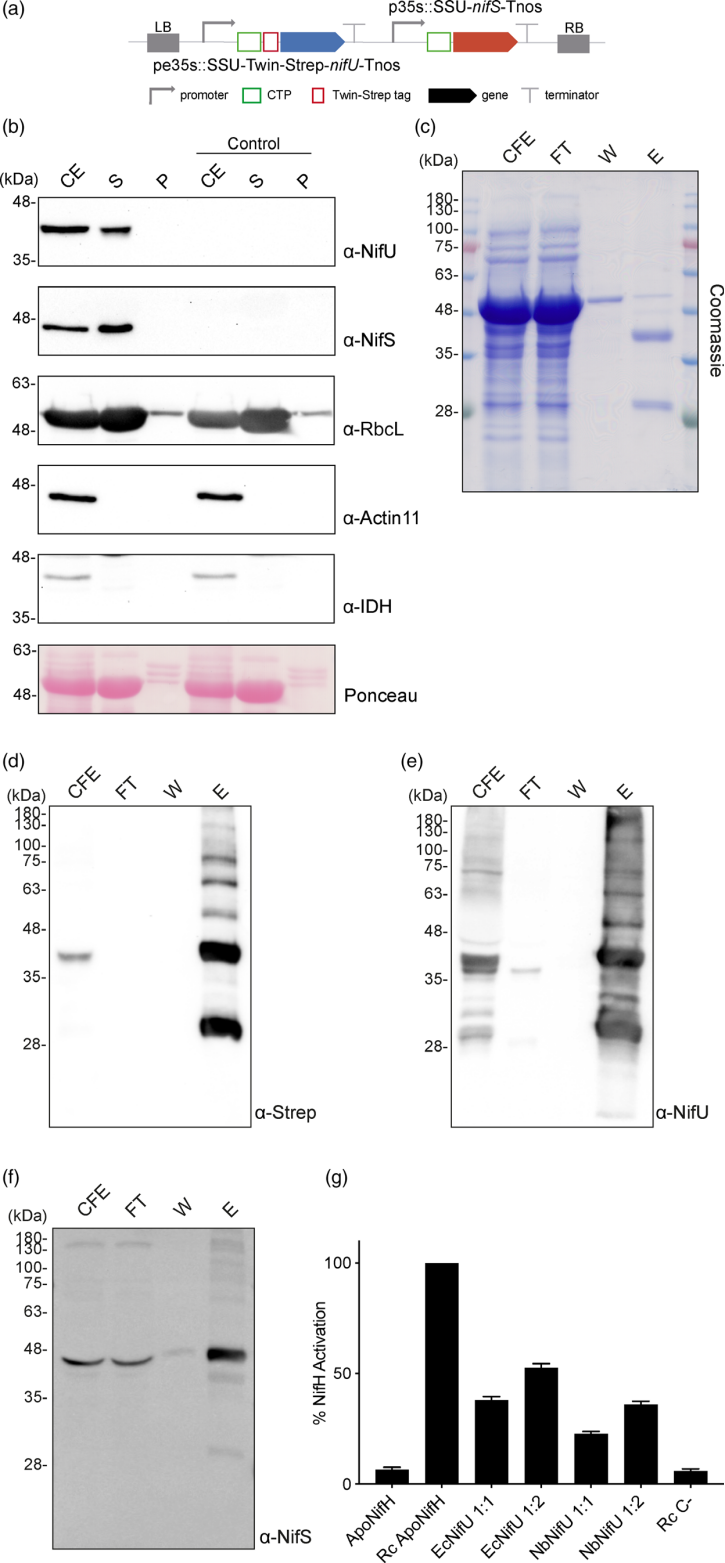
Expression, purification and activity assays of NifU from *N. benthamiana chloroplasts.* (a) Schematic representation of the construct used in *N. benthamiana*‐transient expression assays leading to NifU purification. pe35s: 35S mosaic cauliflower virus‐enhanced promoter; SSU: CTP from the small subunit of RuBisCo; Tnos: nopaline synthase Agrobacterium terminator; p35s: 35S mosaic cauliflower virus promoter. (b) Purification of chloroplasts from NifU and NifS expressing cells. Total proteins extract prior to chloroplast purification (CE). Intact chloroplasts were isolated and then broken to separate the soluble (S) and membrane‐associated (P) fractions. Control refers to *N. benthamiana* plants agroinfiltrated with agrobacterium transformed with an empty vector. Proteins were resolved by SDS‐PAGE and detected by Western blot. Expected sizes are Twin‐Strep NifU: 36.5 kDa, NifS: 43.7 kDa, RbcL: 52.7 kDa; Actin‐11: 41.6 kDa and IDH: 39 kDa. Note that the IDH blot was re‐probed after striping anti‐RuBisco and some signal remains (upper band). (c) Coomassie staining illustrating the Strep‐Tactin purification procedure of *A. vinelandii* NifU expressed in leaves of *N. benthamiana*. CFE, cell‐free extract (soluble fraction following centrifugation and filtering of disrupted leaf tissue); FT, Strep‐Tactin flow‐through fraction; W, wash fraction; E, biotin‐eluted fraction. (d–f) Western blot analysis of the Strep‐Tactin purification procedure. Antibodies targeting Strep‐tag (d) or NifU (e) were used for visualization of the purified NifU protein. Antibodies targeting NifS (f) were used to visualize NifS protein co‐purifying with NifU. (g) *A. vinelandii* apo‐NifH was activated either by *in vitro* [4Fe–4S] cluster reconstitution (Rc Apo‐NifH) or by incubation with cluster‐loaded NifU purified from *E. coli* (EcNifU) or from *N. benthamiana* leaves (NbNifU) in 1:1 or 1:2 NifH/NifU molar ratio. Identical reaction mixtures without NifU were used as negative control (Rc C‐). Error bars represent mean ± SD (*n* = 2).

To further study NifU and NifS functionality, yeast codon‐optimized genes were used to generate a construct in which NifU was tagged with Twin‐Strep‐tag and both genes were fused in their N‐terminal end to the chloroplast targeting sequence SSU for transient expression in *N. benthamiana* leaves (Figure 6a).

Chloroplast purification assays showed that both proteins accumulated at high levels in the stroma (Figure 6b), and NifU could be purified using the Twin‐Strep‐tag. The biotin‐eluted fraction contained two main polypeptides, one migrating at the expected molecular weight of a Twin‐Strep‐tagged full length NifU (around 40 kDa) and another with higher mobility (over 30 kDa). Both isoforms cross‐reacted with anti‐Strep and anti‐NifU antibodies (Figure 6c–e). The faster migration polypeptide was present already in the cell‐free extract, but appeared more abundant after the purification process, consistent with being a degradation product of full length NifU. Mass spectroscopy confirmed it was a truncated NifU protein lacking part of its C‐terminus (Figure S6). A fraction of NifS co‐purified with NifU in the Strep‐Tactin column (Figure 6f), indicating that these two proteins are able to interact in the chloroplast to some degree. The purified NifU prep was light brown and contained 1.5 Fe atoms bound per monomer, consistent with it carrying a permanent [2Fe‐2S] cluster per NifU dimer (Smith et al., 2005) (For the UV‐Vis spectrum of the purified protein, see Figure S7).

When NifU was expressed alone (Figure S8), its Fe content was much lower (0.68 per monomer), suggesting that NifS is active in transferring the sulphur necessary for the assembly of [Fe‐S] clusters. To measure NifU functionality, we tested its ability to deliver these [Fe‐S] clusters to *A. vinelandii* apo‐NifH similar to what was done in López‐Torrejón *et al. *(2016), where recombinant NifU expressed in the yeast mitochondrial matrix was studied. A preparation containing tobacco chloroplast NifU was able to serve as scaffold for the assembly of [Fe‐S] clusters and to transfer them to activate a [4Fe‐4S] cluster‐deficient form of NifH (apo‐NifH). The activities observed for tobacco NifU were around 60% of those of *E. coli* NifU (Figure 6g). Considering that an important fraction of the NifU‐purified preparation consists on the truncated NifU, the activity observed suggests high functionality of the full length protein and supports that the chloroplasts of tobacco leaf cells are also able to accumulate functional NifU. These results, together with the evidence of the accumulation of active NifH, open a feasible avenue of work on plastid engineering for the generation of nitrogen fixing plants.

## Discussion

### Optimization of nif gene synthetic design and chloroplast targeting

The use of *nif* genes synthetically designed to maximize expression in tobacco led to a much higher protein accumulation than the use of the yeast‐optimized versions (Figure 1), although the CAIs for these synthetically designed genes (0.76, 0.77, 0.79 and 0.72 for *nifH, nifM, nifU* and *nifS*, respectively) were in all cases slightly lower than those of the ones optimized for yeast. Adapting the codon usage frequencies of a foreign sequence to that of the host organism was not enough to guarantee high gene expression and protein accumulation, and the advantage obtained by the inclusion of more parameters in the design of the synthetic genes clearly outweighed the penalty of a small decrease in the CAI. More complex approaches, like the one used here, should be considered when the goal is to achieve high levels of recombinant protein. Note that using a complete synthetic design allowed us to obtain NifH protein levels 170 times higher than those achieved by using simply a codon‐optimized sequence (Figure 1d), amounts enough for Fe protein purification and activity tests.

The use of the nuclear transformation approach requires that the protein import into the plastid must be optimized as well. The use of CTPs is a strategy that has already been used in many biotechnological applications to direct recombinant proteins to the chloroplast stroma (Van den Hoppmann *et al.*, 2002; Kavanagh *et al.*, 1988; Kim *et al.*, 2009, 2010; Lee *et al.*, 2008; Broeck *et al.*, 1985; Zhong *et al.*, 2003). However, we found that most of the CTPs described in the literature included a sequence longer than the transit peptide, leaving scar amino acid residues in the N‐terminus of the imported proteins after CTP cleavage. We used bioinformatics tools to predict the minimal sequence required to direct recombinant proteins to the subcellular compartment and that would leave the minimal scar after cleavage (ChloroP 1.1 server (Emanuelsson *et al.*, 1999)). In most cases, the extended version of the CTP did not work better than the minimal version, thus having a longer sequence does not seem beneficial, and only the minimal versions should be used in the future, as they comprise clean and efficient tools for recombinant protein targeting (Figure 2).

As expected, CTPs showed different behaviour when fused to different Nif proteins, highlighting the influence of cargo proteins in import efficiency (Shen *et al.*, 2017). Although some CTPs, like the one from RuBisCo small subunit (*Nt*RBS CTP), seemed to perform well in all four cases (Figure 2), diversifying the CTPs used is desirable for synthetic biology efforts in which multiple *nif* genes are to be co‐expressed. In this case, avoiding the repetition of CTPs would be beneficial since homologous recombination between repeated sequences is a major cause of genomic rearrangements, producing insertion/deletions and translocations.

Targeting of proteins to mitochondria and chloroplasts is generally highly specific, but an increasing number of proteins imported dually into both organelles have been described (Peeters and Small, 2001). In this work, chloroplast purification assays clearly show that the selected CTPs drive Nif proteins primarily to the chloroplast, and microscopy of the GFP fusions confirms specific organelle targeting.

The observation of cleavage of the transit peptide in a Western blot is often indicative of correct localization of the recombinant protein, but should be confirmed. Our results indicate that when expressed alone, NifH is imported into the organelle and the CTP correctly processed. However, NifM co‐expression was necessary to lead to soluble stroma NifH accumulation. With a few exceptions, the production of an active NifH is known to depend on NifM (Howard *et al.*, 1986), and our data suggest that this requirement extends to plant cells.

The observed compartmentalization of NifH in the absence of NifM is reminiscent of the bodies formed in plant cells during piecemeal chlorophagy, a process that involves the formation of small vesicle‐like structures that bud from plastids and are delivered to the vacuole for degradation (Otegui, 2018). All this information suggests improper folding of the NifH protein when expressed alone, that triggers this or another similar degradation pathway within the plant cell.

### Purification of active NifU and NifH from tobacco chloroplasts

The recovery of relatively high Fe protein activity expressed in a plant is a major accomplishment. The activity obtained is still lower than that of endogenous *A. vinelandii* NifH or the one obtained from yeast mitochondria (López‐Torrejón *et al.*, 2016), so future efforts should be directed at fine tuning its maturation and/or preventing damage of its [4Fe‐4S] cluster by O_2_. As NifH is extremely sensitive and irreversibly damaged by O_2_ (Shah *et al.*, 1973), finding a functional form within the chloroplast indicates this organelle is able to provide an anoxygenic (enough) environment for the enzyme, at least during the night period, after which tissue collection took place. This is in accordance with the observation that *C. reinhardtii* mutants in *chlL*, an essential chloroplast gene required for chlorophyll biosynthesis in the dark, that could be partially complemented with the *K. pneumoniaenifH* (Cheng *et al.*, 2005).

Functional NifU was also isolated from plant cells for the first time. In this case, the synthetic design of the genes was not necessary to achieve high enough protein levels to test for its activity; however, it was lower than that of the recombinant NifU purified from yeast mitochondrial matrix (López‐Torrejón *et al.*, 2016). This could be related to the presence of a degradation product lacking at least part of the Nfu‐like C‐terminal domain of NifU (Figure S6), previously shown to participate in the assembly of [4Fe‐4S] clusters (Smith et al., 2005).

The co‐elution of NifU with NifH points to an interaction between these two proteins, suggestive of an actual cluster transfer occurring between the two. The co‐elution of NifM with NifH could be interpreted in the light of NifM‐mediated maturation of NifH. It is possible that in our transient expression system the time in which the two proteins cohabitate within the chloroplast is too short for complete maturation of NifH and in the future it will be important to test whether, in stable transformants, this interaction diminishes leading to a higher activity of (properly matured) NifH.

The lack of NifH activity when it was only co‐expressed with *n* *ifM*, but not *n* *ifU* or *n* *ifS*, suggests that the chloroplast machinery cannot efficiently transfer [4Fe‐4S] clusters to NifH. We cannot completely exclude, however, the possibility that the effect of NifU and NifS is indirect and being asserted by other iron–sulphur protein that in turn has a positive effect on NifH, or by reducing the possible stress caused by excessive free iron. Previously, only a very low and difficult to quantify NifH activity had been detected in transplantomic plants expressing NifH and NifM after incubation of whole plants under a low O_2_ atmosphere (Ivleva *et al.*, 2016). By using a different technical approach and incorporating NifU and NifS, we have obtained significant levels of activity for NifH in plants grown with no manipulation of the atmospheric O_2_ content, demonstrating that the nuclear encoding of plastid‐targeted *nif* genes constitutes a worthy strategy for engineering nitrogenase assembly in the plastids of higher plants.

## Experimental procedures

### Molecular biology

The construct containing SSU‐Twin‐Strep *nifU*/SSU *nifS* was generated from the vector pGFPGUSplus (Addgene #64401, Watertown, Massachusetts, USA). pGFPGUSplus was digested with HindIII and BglII and a synthetic DNA fragment containing the enhanced p35S promoter, the CTP from the RuBisco small subunit of *N. plumbangifolia* SSU (Table S2) and the Twin‐Strep‐tag (Thermo Fisher Scientific, Waltham, MA, USA) was ligated within, generating pN2SB4.

The yeast codon‐optimized *nifU* sequence was amplified using primers 2072 and 2636 (primers sequences in Appendix S1) using pN2GLT4 (López‐Torrejón *et al.*, 2016) as template. Plasmid pN2SB4 was digested with BamHI and BstEII, and the PCR product was ligated to generate pN2XJ196. To add p35s::SSU‐*nifS*, the DNA sequence was amplified from pN2GLT4 using overlapping PCR with the primers 2423, 2430, 2339 and 2431. The amplified fragment and the plasmid pN2XJ196 were digested using SpeI‐SacI and XbaI‐SacI, respectively, and then ligated to generate the final construct.

All other constructs used in this work were generated using the MoClo cloning system (Weber *et al.*, 2011; Werner *et al.*, 2012). For the generation of Level 0 plasmids, the design of primers was performed manually following the rules described in Weber *et al. *(2011). When the fragments contained an internal restriction site for BsaI or BpiI, the piece was domesticated using internal primers to mutate the site. The design of domestication primers was done using the Domesticator Tool (https://gbcloning.upv.es/do/domestication/) subsequently modifying the tails of the primers to add a BpiI restriction site. Modular pieces were amplified by PCR using Phusion Hot Start II DNA Polymerase (Thermo Fisher) with primers and templates described in Appendix S1.

MoClo restriction‐ligations were set up in a final volume of 20 μL with a 2:1 molar ratio of insert:acceptor vector, 5U of the required restriction enzyme – BpiI (Thermo Fisher Scientific, Waltham, MA, USA) for the generation of Level 0 and Level 2 plasmids and BsaI‐HFv2 (NEB) for the generation of Level 1 plasmids – 4.5U of T4 Ligase (Promega, Madison, WI, USA), 1.5 μL of Ligase 10x Buffer and 1.5 μL of 10x BSA (Canvax Biotech, Córdoba, Spain). The reaction was incubated in a thermocycler as follows: 20 s at 37 ºC, [3 min at 37 ºC, 4 min at 16 ºC] for 26 cycles, 5 min at 50 ºC, 5 min at 80 ºC and hold at 16 ºC.

The reaction mix was then added to *Escherichia coli* DH5α chemically competent cells which were grown in LB solid medium containing 20 μg/mL X‐Gal (Duchefa Biochemie, Haarlem, Netherlands) and 1 mm IPTG (Sigma‐Aldrich, Darmstadt, Germany), supplemented with 50 μg/mL spectinomycin (Sigma‐Aldrich, Darmstadt, Germany) for Level 0, 100 μg/mL carbenicillin (Formedium, Norfolk, UK) for Level 1 or kanamycin (Formedium, Norfolk, UK) for Level 2 constructs. White colonies were selected for plasmid DNA extraction using the GenElute Plasmid Miniprep Kit (Sigma‐Aldrich, Darmstadt, Germany). The integrity of all plasmids was verified by Sanger sequencing (Macrogen, Madrid, Spain; Eurofins Genomics, Ebersberg, Germany).

A list of all transcriptional units and multigenic constructs generated and used in this work can be found in Appendices S2 and S3, respectively.

### Transient expression assays

*N. benthamiana* plants were cultivated on 8 x 8 x 8 cm pots in a greenhouse in long‐day conditions (16‐h light/8‐h dark) ensuring enough light with LED lamps at 26/22 ºC day/night temperature.

*Agrobacterium tumefaciens* GV3101 strain cells were transformed with the plasmid of interest and grown at 28 ºC and shaking in LB medium containing rifampicin (25 μg/mL) (Sigma‐Aldrich) and gentamicin (10 μg/mL) (Sigma‐Aldrich), supplemented with selective antibiotics as needed. Overnight cultures were diluted to an OD_600_ between 0.3 and 0.6 in an infiltration solution containing 10 mm MES pH 5.5, 10 mm MgSO_4_ and 150 μm acetosyringone (Sigma‐Aldrich) and incubated at 150 rpm at RT for 3 h. When using single transcriptional units, an Agrobacterium GV3101 strain carrying the silencing suppressor p19 (Li, 2011; Naim *et al.*, 2012) was co‐diluted to an OD_600_ of 0.3‐0.6 (multigenic constructs already contain p19 transcriptional unit). Leaves of 3‐ to 4‐week‐old *N. benthamiana* plants were agroinfiltrated using a syringe without needle, and 3‐4 days after infiltration, tissue was collected at the end of the dark period and used for protein extraction, chloroplast isolation or confocal microscopy (protocols performed under ambient light).

### Analysis of protein expression

Leaf discs from infiltrated *N. benthamiana* plants were harvested into 2‐mL screw‐cap tubes containing 5 3‐mm‐diameter glass beads, and their weight was measured. Tubes were immediately frozen in liquid N_2_ and stored at −80 ºC for further use. Tissue was ground to a fine powder using a BeadBug homogenizer (Benchmark Scientific Inc, Sayreville, NJ, USA) at maximum speed for 45 s avoiding thawing. Samples were mixed with 4 volumes of 2x Laemmli buffer, boiled for 10 min, cooled down and centrifuged at 14 000 *g* for 3 min at 4 ºC to obtain ‘crude extracts (CE)’.

SDS‐PAGE and immunoblot analysis were performed by standard methods. Antibodies used were as follows: polyclonal antibodies detecting NifH, NifU, NifS (López‐Torrejón *et al.*, 2016) and NifM (Burén *et al.*, 2017) were raised against purified preparations of the corresponding *A. vinelandii proteins*. Commercially available antibodies against GFP (11814460001; Roche, Basel, Germany), Strep‐tag II (Strep‐Tactin‐HRP, 2‐1502‐001; IBA Lifesciences, Göttingen, Germany), RbcL (AS03 037; Agrisera AB, Vännäs, Sweden), Actin‐11 (AS10 702; Agrisera AB, Vännäs, Sweden) and IDH (AS06 203A; Agrisera AB, Vännäs, Sweden) were used. Secondary antibodies, horseradish peroxidase‐conjugated anti‐rabbit IgG (A0545‐1ML; Sigma‐Aldrich) or anti‐mouse IgG (AS11 1772; Agrisera AB, Vännäs, Sweden), were used as appropriate.

Immunoblots were developed in an iBright FL1000 Imaging System (Thermo Fisher) using chemiluminescence mode. *Smart exposure* setting was used for all blots except for those in Figure 3, where the *Signal accumulation* setting was used. Processing of the images was performed with iBright Analysis Cloud Service (Thermo Fisher), applied equally across the entire image as well as to controls.

For relative protein accumulation measurements, short exposure blots were exported for analysis from iBright FL1000 (Thermo Fisher) as TIFF files and the signal intensity for each band was measured with Image Studio Lite (LI‐COR Biosciences, Lincoln, Nebraska, USA). The given value for each Nif protein band was normalized using the value corresponding to GFP in the same lane.

### Confocal microscopy

Leaf discs from 4‐ to 5‐week‐old plants were imaged using a Zeiss LSM880 confocal laser scanning microscope (Zeiss, Oberkochen, Germany) equipped with a Plan‐Apochromat 40X/1.2 water‐immersion objective and ZEN 2.6 Black software (Zeiss, Oberkochen, Germany). The excitation laser lines wavelength/emission bands used were as follows: GFP (488 nm/493 to 556 nm), BFP (405 nm/410 to 529 nm), mCherry (561 nm/595 to 640 nm) and chlorophyll autofluorescence (633 nm/647 to 721 nm). To avoid overlap between the fluorescence channels, sequential scanning was used when necessary. Images were processed with ZEN 2.6 (Zeiss, Oberkochen, Germany) and assembled using Illustrator software (Adobe, San José, CA, USA).

### Chloroplast isolation assays

Isolation of chloroplasts from agroinfiltrated leaves was performed as described in Klinkenberg (2014), with slight modifications. Plant material was harvested and homogenized in chloroplast isolation buffer (CIB) with the help of a Potter homogenizer. After filtration through a 47 μm nylon mesh, the intact chloroplasts were separated by centrifugation through a double Percoll (GE Healthcare, Chicago, IL, USA) gradient (40/80%) at 3200 *g* at 4 °C for 15 min. The band formed at the interface was collected and washed twice with CIB without BSA. Chloroplasts were shock frozen, re‐suspended in ice‐cold protein extraction buffer and centrifuged at 15 000 *g* at 4 °C for 10 min. The supernatant ‘stroma fraction (S)’ was recovered, and the ‘pellet fraction (P)’ was re‐suspended in the same volume of protein extraction buffer.

### NifU purification

Agroinfiltrated *N. benthamiana* leaves were frozen in liquid nitrogen and processed using the Oster Classic blender 4655 inside an anaerobic Coy Laboratory Products glovebox. Leaves were homogenized with lysis buffer (Tris–HCl 100 mm pH 8.6, NaCl 200 mm, glycerol 10%, PMSF 1 mm, leupeptin 1 μg/mL, DNAseI 5 μg/mL and 200 μL of Biolock (IBA Lifesciences) in a 1:1 ratio. The extract was filtered through a cloth and then centrifuged for 36 200 *g* during 1 h at 4 ºC, and the supernatant was filtered through a Nalgene filter unit with 0.2 μm pore size, resulting in the cell‐free extract (CFE).

Twin‐Strep‐tag NifU was purified by Strep‐Tactin XT affinity chromatography under anaerobic conditions (<0.1 ppm O_2_) using an AKTA Prime FPLC system (GE Healthcare, Chicago, IL, USA) inside an MBraun glovebox. The CFE was loaded into a 5‐mL Strep‐Tactin‐XT high capacity cartridge (IBA Lifesciences) at a 2.5 mL/min flow after equilibration with 20 column volumes of Wash Buffer (100 mm Tris–HCl pH 8.6, 200 mm NaCl, 10% glycerol) and then washed with 15 column volumes of Wash Buffer and eluted using 50 mm biotin (IBA Lifesciences) in Wash Buffer. The protein was concentrated down to 1 mL using an Amicon Ultra Centrifugal Filter unit (Merck Millipore, Burlington, MA, USA) of 30 kDa cut‐off and desalted using a Sephadex G‐25 PD‐10 column (GE Healthcare, Chicago, IL, USA) to remove biotin. The elution of the PD‐10 column was concentrated to 500 μL and stored in cryogenic tubes (Nalgene, Rochester, NY, USA) in liquid nitrogen.

### Iron content measurement

The iron content of purified NifU protein was determined by the colorimetric method described in Fish (1988).

### In vitro apo‐NifH reconstitution assays using NifU isolated from E. coli and N. benthamiana

Apo‐NifH was prepared *in vitro* according to Rangaraj *et al. *(1997), with slight modifications. NifH (50 µm) purified from *A. vinelandii* was incubated with 2.5 mm MgATP, 2 mm DTH and 40 mm 2,2′‐bipyridyl in 22 mm Tris–HCl (pH 7.4) for 30 min at 25 °C. The apo‐NifH protein was desalted twice using Sephadex G‐25 PD‐10 columns (GE Healthcare, Chicago, IL, USA) and eluted in buffer containing 200 mm NaCl, 10% glycerol, 2 mm DTH and 100 mm Tris–HCl (pH 8.0). Final protein was concentrated using Amicon Ultra 30K centrifugal filters (Merck Millipore, Burlington, MA, USA), and protein concentration was determined using the BCA protein assay (Thermo Fisher Scientific, Waltham, MA, USA).

*In vitro* [4Fe–4S] cluster reconstitution was performed essentially as described by López‐Torrejón *et al. *(2016). [Fe–S] clusters were assembled on apo‐NifH (Rc Apo‐NifH) or NifU (isolated from *E. coli* (López‐Torrejón *et al.*, 2016) or *N. benthamiana* (agroinfiltration of pN2XJ196)) according to (Dos Santos *et al.*, 2004), with modifications. The cluster assembly mixture included apo‐NifH, *E. coli* or *N. benthamiana* NifU (20 µm), 9 mm DTT, 0.4 mm (NH_4_)_2_Fe(SO_4_)_2_, 1 mm l‐cysteine, 225 nm NifS and 22 mm Tris–HCl (pH 7.4). Reactions were incubated for 3 h on ice. After the cluster assembly, the buffer of mixtures was exchanged to remove DTT and the excess of Fe and L‐cysteine.

For NifU to apo‐NifH cluster transfer [4Fe–4S], cluster assembly mixtures were prepared with increasing concentrations of NifU (1:1 and 1:2 NifH/NifU molar ratios) prior to the addition of 0.862 µm apo‐NifH in a final volume of 300 µL. Cluster transfer was allowed in 22 mm Tris–HCl (pH 7.4) and 4 mm DTH before addition of 300 µL ATP‐regenerating mixture (1.23 mm ATP, 18 mm phosphocreatine disodium salt, 2.2 mm MgCl_2_, 3 mm DTH and 40 μg/mL creatine phosphokinase in 22 mm Tris–HCl buffer (pH 7.4), final concentrations) and pure *A. vinelandii* NifDK (to obtain a 20x Fe/MoFe protein molar ratio). Acetylene reduction assays were performed in 9‐mL vials under Ar atmosphere at 30 °C for 15 min following standard procedures (Shah *et al.*, 1973). To establish the 100% NifH protein‐specific activity under the same reaction conditions, control reactions with purified holo‐NifH were used.

### NifH purification

Agroinfiltrated *N. benthamiana* fresh leaves were ground in liquid N_2_ in the presence of sea sand and re‐suspended in anaerobic lysis buffer (100 mm Tris–HCl pH 8.0, 250 mm NaCl) containing 1 mm phenylmethylsulfonyl fluoride (PMSF), 1 mm leupeptin, 2 mm sodium dithionite (DTH) and 5 µg/mL DNaseI. After blender homogenization, the plant solution was centrifuged at 73 000 *g* for 1 h at 4 ºC under anaerobic conditions and cell‐free extract was obtained.

Twin‐Strep‐tag NifH was purified by Strep‐Tactin XT affinity chromatography under anaerobic conditions (<0.1 ppm O_2_) using an AKTA Prime FPLC system (GE Healthcare, Chicago, IL, USA) inside an MBraun glovebox. Total protein extract was loaded at 2 mL/min into a 5‐mL Strep‐Tactin‐XT high capacity cartridge (IBA Lifesciences) equilibrated with lysis buffer 2 mm DTH, washed with 20 column volumes of lysis buffer 2 mm DTH and eluted with six column volumes of elution buffer 2 mm DTH (50 mm Tris–HCl pH 8.0, 250 mm NaCl, 5% glycerol, 50 mm biotin). The elution fraction was concentrated with cut‐off pore size of 30 kDa using a centrifugal membrane device (Amicon Ultra‐15; Millipore) and then desalted in a PD‐10 desalting column (GE Healthcare, Chicago, IL, USA) equilibrated with desalting buffer (50 mm Tris–HCl, pH 8, 300 mm NaCl) containing 2 mm DTH. Purified NifH was frozen and stored in cryogenic tubes (Nalgene, Rochester, NY, USA) in liquid nitrogen, until used.

### Fe protein activity determination

Fe protein activity of NifH preparations purified from *N. benthamiana* was analysed by the acetylene reduction assay after addition of 13 μm MoFe protein and ATP‐regenerating mixture (1.23 mm ATP, 18 mm phosphocreatine, 2.2 mm MgCl_2_, 3 mm DTH and 40 µg of creatine phosphokinase) (Shah and Brill, 1973). Positive control reactions were carried out with Fe protein and MoFe proteins purified from *A. vinelandii* under anaerobic conditions as described (Curatti *et al.*, 2007).

## Conflict of interest

The authors have no conflict of interest to report.

## Author contributions

LMR, AE and EC designed the experiments. XJ cloned and expressed *nifU* and *nifS*, and purified NifU. NSB conducted the NifU activity assays. GLT and AE purified NifH. GLT conducted NifH activity assays. AE performed the rest of the experiments. EC wrote the paper.

## Data deposition

The complete nucleotide sequences of the *nifH, nifM, nifU* and *nifS* yeast codon‐optimized genes and tobacco synthetic designed genes have been deposited into GenBank under the accession numbers MN920692 to MN920699.

## Supporting information


**Figure S1** Mass spectrometry results of bands present in NifH purification.
**Figure S2** Determination of N‐terminal sequence of Twin‐Strep‐NifH using the standard Edman degradation method.
**Figure S3** NifM and NifU co‐elution of with Strep‐tagged NifH.
**Figure S4** Confirmation of the activity of N‐terminal Strep‐tagged *A. vinelandii* NifH protein.
**Figure S5** Purification of NifH from chloroplasts of *N. benthamiana* co‐expressing nifH and nifM.
**Figure S6** Mass spectrometry results of full length and truncated yeast codon‐optimized NifU expressed in *N. benthamiana*.
**Figure S7** UV‐Vis spectrum of yeast codon‐optimized NifU expressed in *N. benthamiana*.
**Figure S8** Purification of NifU from chloroplasts of *N. benthamiana*.

**Table S1** Optimization and motif settings used in Codon Optimization OnLine for nif synthetic designed genes.
**Table S2** Summary of Chloroplast Transit Peptides studied in this work.

**Appendix S1** List of modular pieces and primers used.
**Appendix S2** List of assembled Transcriptional Units.
**Appendix S3** List of multigenic constructions.
**Appendix S4** Experimental Procedures from the data shown in Supporting Figures.
