# Supplementary material for: Use of synthetic biology tools to optimize the production of active nitrogenase Fe protein in chloroplasts of tobacco leaf cells
Source: Plant Biotechnol J. 2020 Apr 7;18(9):1882–96. doi: 10.1111/pbi.13347 (PMC7415783; doi:10.1111/pbi.13347)

Figure S1. Mass spectrometry results of bands present in NifH purification.

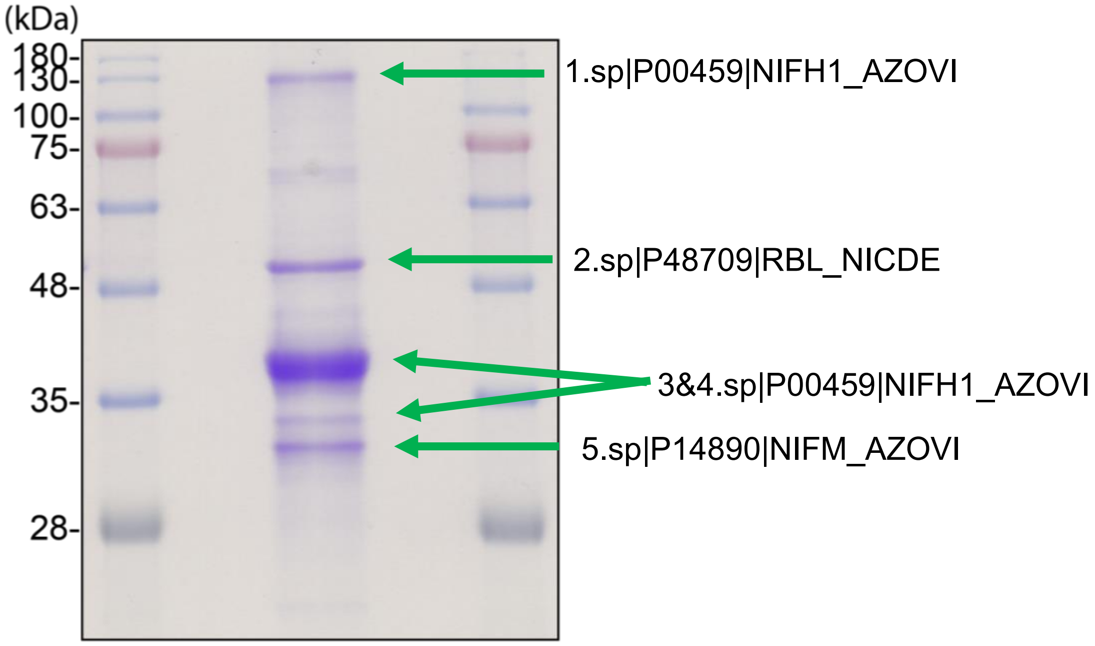

Estimation of the purity of NifH preparation expressed in *N. benthamiana.* The band corresponding to the full-size NifH protein amounts for 59,05% of the total protein present in the preparation, quantified using ImageJ (Schneider *et al.*, 2012).

Peptide mass fingerprint Mascot search results against SwissProt database

Band 1

Match to: **NIFH1_AZOVI** Score: **218** Expect: **8.8e-017**
Nitrogenase iron protein 1 OS=Azotobacter vinelandii GN=nifH1 PE=1 SV=2

Nominal mass (M_r): **31895**; Calculated pI value: **4.68**

Fixed modifications: Carbamidomethyl (C)
Variable modifications: Oxidation (M)
Cleavage by Trypsin: cuts C-term side of KR unless next residue is P
Number of mass values searched: **65**
Number of mass values matched: **31**
Sequence Coverage: **63%**

Matched peptides shown in **Bold Red**

1 MAMRQCAIYG KGGIGK**STTT QNLVAALAEM GKKVMIVGCD PKADSTRLIL**
51 **HSKAQNTIME MAAEAGTVED LELEDVLKAG YGGVKCVESG GPEPGVGCAG**
101 **R**GVITAINFL EEEGAYEDDL DFVFYDVLGD VVCGGFAMPI RENK**AQEIYI**
151 **VCSGEMMAMY AANNISKGIV KYANSGSVRL GGLICNSRNT DREDELIIAL**
201 **ANKLGTQMIH FVPRDNVVQR** AEIR**RMTVIE YDPKAKQADE YRALAR**KVVD
251 NKLLVIPNPI TMDELEELLM EFGIMEVEDE SIVGKTAEEV

Start - End	Observed	Mr(expt)	Mr(calc)	ppm	Miss	Sequence
17 - 32	1634.8175	1633.8102	1633.8345	-14.9	0	K.STTTQNLVAALAEMGK.K
17 - 32	1650.8029	1649.7956	1649.8294	-20.5	0	K.STTTQNLVAALAEMGK.K + Oxidation (M)
17 - 33	1762.9146	1761.9073	1761.9295	-12.6	1	K.STTTQNLVAALAEMGKK.V
17 - 33	1778.9027	1777.8954	1777.9244	-16.3	1	K.STTTQNLVAALAEMGKK.V + Oxidation (M)
34 - 42	1018.5150	1017.5077	1017.4987	8.84	0	K.VMIVGCDPK.A
34 - 47	1548.7284	1547.7211	1547.7436	-14.5	1	K.VMIVGCDPKADSTR.L
34 - 47	1564.7222	1563.7149	1563.7385	-15.1	1	K.VMIVGCDPKADSTR.L + Oxidation (M)
48 - 78	3411.9492	3410.9419	3410.7312	61.8	1	R.LILHSKAQNTIMEMAAEAGTVEDLELEDVLK.A
54 - 78	2720.3689	2719.3616	2719.2932	25.2	0	K.AQNTIMEMAAEAGTVEDLELEDVLK.A
54 - 78	2736.3696	2735.3623	2735.2881	27.1	0	K.AQNTIMEMAAEAGTVEDLELEDVLK.A + Oxidation (M)
54 - 78	2752.3582	2751.3509	2751.2830	24.7	0	K.AQNTIMEMAAEAGTVEDLELEDVLK.A + 2 Oxidation (M)
54 - 85	3352.8481	3351.8408	3351.6214	65.5	1	K.AQNTIMEMAAEAGTVEDLELEDVLKAGYGGVK.C
86 - 101	1588.6600	1587.6527	1587.6770	-15.3	0	K.CVESGGPEPGVGCAGR.G
145 - 167	2594.2190	2593.2117	2593.1684	16.7	0	K.AQEIYIVCSGEMMAMYAANNISK.G
145 - 167	2610.2148	2609.2075	2609.1633	16.9	0	K.AQEIYIVCSGEMMAMYAANNISK.G + Oxidation (M)
145 - 167	2626.2317	2625.2244	2625.1583	25.2	0	K.AQEIYIVCSGEMMAMYAANNISK.G + 2 Oxidation (M)
168 - 179	1250.6826	1249.6753	1249.6779	-2.04	1	K.GIVKYANSGSVR.L
172 - 179	853.4413	852.4340	852.4090	29.4	0	K.YANSGSVR.L
180 - 188	989.5306	988.5234	988.5124	11.1	0	R.LGGLICNSR.N
189 - 203	1714.8787	1713.8714	1713.8897	-10.7	1	R.NTDREDELIIALANK.L
204 - 214	1298.6937	1297.6864	1297.6965	-7.78	0	K.LGTQMIHFVPR.D
204 - 214	1314.6843	1313.6770	1313.6914	-11.0	0	K.LGTQMIHFVPR.D + Oxidation (M)
204 - 220	2010.0455	2009.0382	2009.0629	-12.3	1	K.LGTQMIHFVPRDNVVQR.A
204 - 220	2026.0441	2025.0368	2025.0578	-10.4	1	K.LGTQMIHFVPRDNVVQR.A + Oxidation (M)
225 - 234	1251.6332	1250.6259	1250.6329	-5.57	1	R.RMTVIEYDPK.A
225 - 234	1267.6246	1266.6173	1266.6278	-8.28	1	R.RMTVIEYDPK.A + Oxidation (M)
226 - 234	1095.5450	1094.5377	1094.5318	5.43	0	R.MTVIEYDPK.A
226 - 236	1294.6548	1293.6475	1293.6638	-12.6	1	R.MTVIEYDPKAK.Q
226 - 236	1310.6514	1309.6441	1309.6588	-11.2	1	R.MTVIEYDPKAK.Q + Oxidation (M)
235 - 242	980.4969	979.4896	979.4723	17.7	1	K.AKQADEYR.A
237 - 246	1192.6011	1191.5938	1191.5996	-4.84	1	K.QADEYRALAR.K

Band 2

Match to: **RBL_NICDE** Score: **315** Expect: **1.7e-026**
Ribulose bisphosphate carboxylase large chain OS=Nicotiana debneyi GN=rbcL PE=3 SV=1
Nominal mass (M_r): **53316**; Calculated pI value: **6.55**

Fixed modifications: Carbamidomethyl (C)
Variable modifications: Oxidation (M)
Cleavage by Trypsin: cuts C-term side of KR unless next residue is P
Number of mass values searched: **65**
Number of mass values matched: **32**
Sequence Coverage: **52%**

Matched peptides shown in **Bold Red**

1 MSPQTETKAS VGFKAGVK**EY KLTYYTPEYQ TKDTDILAAF R**VTPQPGVPP
51 EEAGAAVAAE SSTGTWTTVW TDGLTSLDRY KGR**CYRIER**V VGEKDQYIAY
101 VAYPLDLFEE GSVTNMFTSI VGNVFGFK**AL RALRLEDLRI PPAYVKTFQG**
151 **PPHGIQVERD K**LNK**YGRPLL GCTIKPK**LGL SAKNYGR**AVY ECLRGGLDFT**
201 **KDDENVNSQP FMR**WR**DRFLF CAEALFK**AQV ETGEIK**GHYL NATAGTCEEM**
251 **IKR**AVFAR**EL GVPIVMHDYL TGGFTANTSL AHYCRDNGLL LHIHRAMHAV**
301 **IDRQKNHGIH FR**VLAKALR**M SGGDHIHSGT VVGKLEGERD ITLGFVDLLR**
351 **DDFVEQDRS**R GIYFTQDWVS LPGVLPVASG GIHVWHMPAL TEIFGDDSVL
401 QFGGGTLGHP WGNAPGAVAN RVALEACVKA R**NEGRDLAQE GNQIIR**EASK
451 WSPELAAACE VWK**EIVFNFA AVDVLDK**

Start - End	Observed	Mr(expt)	Mr(calc)	ppm	Miss	Sequence
19 - 32	1826.8606	1825.8533	1825.8774	-13.2	1	K.EYKLTYYTPEYQTK.D
22 - 32	1406.6692	1405.6619	1405.6765	-10.4	0	K.LTYYTPEYQTK.D
22 - 41	2409.2219	2408.2146	2408.1900	10.2	1	K.LTYYTPEYQTKDTDILAAFR.V
33 - 41	1021.5566	1020.5493	1020.5240	24.8	0	K.DTDILAAFR.V
84 - 89	896.4885	895.4813	895.4334	53.4	1	R.CYRIER.V
132 - 139	985.6098	984.6025	984.5716	31.4	1	R.ALRLEDLR.I
135 - 146	1413.7994	1412.7921	1412.8027	-7.50	1	R.LEDLRIPPAYVK.T
147 - 159	1465.7362	1464.7289	1464.7474	-12.6	0	K.TFQGPPHGIQVER.D
147 - 161	1708.8497	1707.8424	1707.8693	-15.7	1	K.TFQGPPHGIQVERDK.L
165 - 177	1502.8287	1501.8214	1501.8439	-15.0	0	K.YGRPLLGCTIKPK.L
188 - 194	910.4882	909.4809	909.4378	47.4	0	R.AVYECLR.G
195 - 213	2169.9834	2168.9761	2168.9797	-1.64	1	R.GGLDFTKDDENVNSQPFMR.W
195 - 213	2185.9893	2184.9820	2184.9746	3.39	1	R.GGLDFTKDDENVNSQPFMR.W + Oxidation (M)
202 - 213	1451.6112	1450.6039	1450.6147	-7.42	0	K.DDENVNSQPFMR.W
216 - 227	1516.7455	1515.7382	1515.7544	-10.7	1	R.DRFLFCAEALFK.A
218 - 227	1245.6310	1244.6237	1244.6264	-2.12	0	R.FLFCAEALFK.A
237 - 253	1950.9048	1949.8975	1949.9087	-5.75	1	K.GHYLNATAGTCEEMIKR.A
237 - 253	1966.9031	1965.8958	1965.9037	-3.98	1	K.GHYLNATAGTCEEMIKR.A + Oxidation (M)
259 - 285	3022.6309	3021.6236	3021.4477	58.2	0	R.ELGVPIVMHDYLTGGFTANTSLAHYCR.D
259 - 285	3038.6343	3037.6270	3037.4426	60.7	0	R.ELGVPIVMHDYLTGGFTANTSLAHYCR.D + Oxidation (M)
286 - 295	1187.6713	1186.6640	1186.6571	5.86	0	R.DNGLLLHIHR.A
296 - 303	912.5040	911.4968	911.4647	35.2	0	R.AMHAVIDR.Q
296 - 305	1168.5988	1167.5915	1167.6182	-22.9	1	R.AMHAVIDRQK.N
304 - 312	1136.6182	1135.6109	1135.5999	9.71	1	R.QKNHGIHFR.V
306 - 312	880.4976	879.4903	879.4464	49.9	0	K.NHGIHFR.V
320 - 339	2082.0024	2080.9951	2080.9960	-0.43	1	R.MSGGDHIHSGTVVGKLEGER.D + Oxidation (M)
335 - 350	1845.9656	1844.9583	1844.9996	-22.4	1	K.LEGERDITLGFVDLLR.D
340 - 350	1261.7120	1260.7047	1260.7078	-2.44	0	R.DITLGFVDLLR.D
340 - 358	2266.1362	2265.1289	2265.1277	0.52	1	R.DITLGFVDLLRDDFVEQDR.S
432 - 446	1712.8455	1711.8382	1711.8601	-12.8	1	R.NEGRDLAQEGNQIIR.E
436 - 446	1256.6583	1255.6510	1255.6520	-0.81	0	R.DLAQEGNQIIR.E
464 - 477	1579.8053	1578.7980	1578.8294	-19.8	0	K.EIVFNFAAVDVLDK.-

Band 3

Match to: **NIFH1_AZOVI** Score: **152** Expect: **3.5e-010**
Nitrogenase iron protein 1 OS=Azotobacter vinelandii GN=nifH1 PE=1 SV=2

Nominal mass (M_r): **31895;** Calculated pI value: **4.68**

Fixed modifications: Carbamidomethyl (C)
Variable modifications: Oxidation (M)
Cleavage by Trypsin: cuts C-term side of KR unless next residue is P
Number of mass values searched: **65**
Number of mass values matched: **25**
Sequence Coverage: **50%**

Matched peptides shown in **Bold Red**

1 MAMRQCAIYG KGGIGK**STTT QNLVAALAEM GKK**VMIVGCD PKADSTRLIL
51 HSK**AQNTIME MAAEAGTVED LELEDVLKAG YGGVK**CVESG GPEPGVGCAG
101 RGVITAINFL EEEGAYEDDL DFVFYDVLGD VVCGGFAMPI RENK**AQEIYI**
151 **VCSGEMMAMY AANNISKGIV KYANSGSVRL GGLICNSRNT DREDELIIAL**
201 **ANKLGTQMIH FVPRDNVVQR** AEIR**RMTVIE YDPKAKQADE YRALAR**KVVD
251 NKLLVIPNPI TMDELEELLM EFGIMEVEDE SIVGKTAEEV

Start - End	Observed	Mr(expt)	Mr(calc)	ppm	Miss	Sequence
17 - 32	1634.7944	1633.7871	1633.8345	-29.0	0	K.STTTQNLVAALAEMGK.K
17 - 33	1762.8884	1761.8811	1761.9295	-27.4	1	K.STTTQNLVAALAEMGKK.V
17 - 33	1778.8793	1777.8720	1777.9244	-29.4	1	K.STTTQNLVAALAEMGKK.V + Oxidation (M)
54 - 78	2720.2229	2719.2156	2719.2932	-28.5	0	K.AQNTIMEMAAEAGTVEDLELEDVLK.A
54 - 78	2736.2214	2735.2141	2735.2881	-27.0	0	K.AQNTIMEMAAEAGTVEDLELEDVLK.A + Oxidation (M)
54 - 78	2752.2166	2751.2093	2751.2830	-26.8	0	K.AQNTIMEMAAEAGTVEDLELEDVLK.A + 2 Oxidation (M)
54 - 85	3352.5371	3351.5298	3351.6214	-27.3	1	K.AQNTIMEMAAEAGTVEDLELEDVLKAGYGGVK.C
54 - 85	3384.5283	3383.5210	3383.6112	-26.7	1	K.AQNTIMEMAAEAGTVEDLELEDVLKAGYGGVK.C + 2 Oxidation(M)
145 - 167	2594.0999	2593.0926	2593.1684	-29.2	0	K.AQEIYIVCSGEMMAMYAANNISK.G
145 - 167	2610.0969	2609.0896	2609.1633	-28.3	0	K.AQEIYIVCSGEMMAMYAANNISK.G + Oxidation (M)
145 - 167	2626.0823	2625.0750	2625.1583	-31.7	0	K.AQEIYIVCSGEMMAMYAANNISK.G + 2 Oxidation (M)
168 - 179	1250.6654	1249.6581	1249.6779	-15.8	1	K.GIVKYANSGSVR.L
172 - 179	853.3817	852.3744	852.4090	-40.5	0	K.YANSGSVR.L
180 - 188	989.4786	988.4714	988.5124	-41.5	0	R.LGGLICNSR.N
189 - 203	1714.8528	1713.8455	1713.8897	-25.8	1	R.NTDREDELIIALANK.L
204 - 214	1298.6680	1297.6607	1297.6965	-27.6	0	K.LGTQMIHFVPR.D
204 - 214	1314.6600	1313.6527	1313.6914	-29.5	0	K.LGTQMIHFVPR.D + Oxidation (M)
204 - 220	2010.0171	2009.0098	2009.0629	-26.4	1	K.LGTQMIHFVPRDNVVQR.A
204 - 220	2026.0066	2024.9993	2025.0578	-28.9	1	K.LGTQMIHFVPRDNVVQR.A + Oxidation (M)
225 - 234	1251.6139	1250.6066	1250.6329	-21.0	1	R.RMTVIEYDPK.A
225 - 234	1267.6006	1266.5933	1266.6278	-27.2	1	R.RMTVIEYDPK.A + Oxidation (M)
226 - 234	1095.5027	1094.4954	1094.5318	-33.2	0	R.MTVIEYDPK.A
226 - 236	1294.6339	1293.6266	1293.6638	-28.8	1	R.MTVIEYDPKAK.Q
235 - 242	980.4462	979.4390	979.4723	-34.0	1	K.AKQADEYR.A
237 - 246	1192.5792	1191.5719	1191.5996	-23.2	1	K.QADEYRALAR.K

Band 4

Match to: **NIFH1_AZOVI** Score: **173** Expect: **2.8e-012**

Nitrogenase iron protein 1 OS=Azotobacter vinelandii GN=nifH1 PE=1 SV=2

Nominal mass (M_r): **31895**; Calculated pI value: **4.68**

Fixed modifications: Carbamidomethyl (C)
Variable modifications: Oxidation (M)
Cleavage by Trypsin: cuts C-term side of KR unless next residue is P
Number of mass values searched: **65**
Number of mass values matched: **28**
Sequence Coverage: **59%**

Matched peptides shown in **Bold Red**

1 MAMRQCAIYG KGGIGK**STTT QNLVAALAEM GKKVMIVGCD PKADSTR**LIL
51 HSK**AQNTIME MAAEAGTVED LELEDVLKAG YGGVKCVESG GPEPGVGCAG**
101 **R**GVITAINFL EEEGAYEDDL DFVFYDVLGD VVCGGFAMPI RENK**AQEIYI**
151 **VCSGEMMAMY AANNISK**GIV K**YANSGSVRL GGLICNSRNT DREDELIIAL**
201 **ANKLGTQMIH FVPRDNVVQR** AEIR**RMTVIE YDPKAKQADE YRALAR**KVVD
251 NKLLVIPNPI TMDELEELLM EFGIMEVEDE SIVGKTAEEV

Start - End	Observed	Mr(expt)	Mr(calc)	ppm	Miss	Sequence
17 - 32	1634.8141	1633.8068	1633.8345	-16.9	0	K.STTTQNLVAALAEMGK.K
17 - 32	1650.8110	1649.8037	1649.8294	-15.6	0	K.STTTQNLVAALAEMGK.K + Oxidation (M)
17 - 33	1762.9119	1761.9046	1761.9295	-14.1	1	K.STTTQNLVAALAEMGKK.V
17 - 33	1778.9048	1777.8975	1777.9244	-15.1	1	K.STTTQNLVAALAEMGKK.V + Oxidation (M)
34 - 42	1018.5162	1017.5089	1017.4987	10.0	0	K.VMIVGCDPK.A
34 - 47	1548.7299	1547.7226	1547.7436	-13.6	1	K.VMIVGCDPKADSTR.L
34 - 47	1564.7418	1563.7345	1563.7385	-2.56	1	K.VMIVGCDPKADSTR.L + Oxidation (M)
54 - 78	2720.3606	2719.3533	2719.2932	22.1	0	K.AQNTIMEMAAEAGTVEDLELEDVLK.A
54 - 78	2736.3652	2735.3579	2735.2881	25.5	0	K.AQNTIMEMAAEAGTVEDLELEDVLK.A + Oxidation (M)
54 - 78	2752.3560	2751.3487	2751.2830	23.9	0	K.AQNTIMEMAAEAGTVEDLELEDVLK.A + 2 Oxidation (M)
54 - 85	3352.8301	3351.8228	3351.6214	60.1	1	K.AQNTIMEMAAEAGTVEDLELEDVLKAGYGGVK.C
54 - 85	3368.8384	3367.8311	3367.6163	63.8	1	K.AQNTIMEMAAEAGTVEDLELEDVLKAGYGGVK.C + Oxidation (M)
86 - 101	1588.6731	1587.6658	1587.6770	-7.04	0	K.CVESGGPEPGVGCAGR.G
145 - 167	2594.2185	2593.2112	2593.1684	16.5	0	K.AQEIYIVCSGEMMAMYAANNISK.G
145 - 167	2610.2239	2609.2166	2609.1633	20.4	0	K.AQEIYIVCSGEMMAMYAANNISK.G + Oxidation (M)
145 - 167	2626.2085	2625.2012	2625.1583	16.4	0	K.AQEIYIVCSGEMMAMYAANNISK.G + 2 Oxidation (M)
172 - 179	853.4465	852.4392	852.4090	35.5	0	K.YANSGSVR.L
180 - 188	989.5368	988.5295	988.5124	17.3	0	R.LGGLICNSR.N
189 - 203	1714.8777	1713.8704	1713.8897	-11.2	1	R.NTDREDELIIALANK.L
204 - 214	1298.6947	1297.6874	1297.6965	-7.01	0	K.LGTQMIHFVPR.D
204 - 214	1314.6890	1313.6817	1313.6914	-7.39	0	K.LGTQMIHFVPR.D + Oxidation (M)
204 - 220	2010.0569	2009.0496	2009.0629	-6.61	1	K.LGTQMIHFVPRDNVVQR.A
204 - 220	2026.0487	2025.0414	2025.0578	-8.10	1	K.LGTQMIHFVPRDNVVQR.A + Oxidation (M)
225 - 234	1251.6360	1250.6287	1250.6329	-3.33	1	R.RMTVIEYDPK.A
225 - 234	1267.6428	1266.6355	1266.6278	6.09	1	R.RMTVIEYDPK.A + Oxidation (M)
226 - 234	1095.5449	1094.5376	1094.5318	5.34	0	R.MTVIEYDPK.A
226 - 236	1294.6613	1293.6540	1293.6638	-7.59	1	R.MTVIEYDPKAK.Q
237 - 246	1192.6123	1191.6050	1191.5996	4.56	1	K.QADEYRALAR.K

Band 5

Match to: **NIFM_AZOVI** Score: **70** Expect: **0.055**
Putative peptidyl-prolyl cis-trans isomerase NifM OS=Azotobacter vinelandii GN=nifM PE=3 SV=1

Nominal mass (M_r): **33181**; Calculated pI value: **5.45**
Fixed modifications: Carbamidomethyl (C)
Variable modifications: Oxidation (M)
Cleavage by Trypsin: cuts C-term side of KR unless next residue is P
Number of mass values searched: **65**
Number of mass values matched: **10**
Sequence Coverage: **39%**
Matched peptides shown in **Bold Red**

1 MASER**LADGD SRYYLLK**VAH EQFGCAPGEL SEDQLQQADR IIGR**QRHIED**
51 **AVLRSPDAIG VVIPPSQLEE AWAHIASRYE SPEALQQALD AQALDAAGMR**
101 **AMLAR**ELRVE AVLDCVCAGL PEISDTDVSL YYFNHAEQFK VPAQHKAHIL
151 VTINEDFPEN TREAAR**TRIE TILK**RLRGKP ERFAEQAMKH SECPTAMQGG
201 LLGEVVPGTL YPELDACLFQ MAR**GELSPVL ESPIGFHVLY CESVSPARQL**
251 **TLEEILPR**LR DRLQLRQRKA YQRKWLVCLL QQNATLENLA HG

Start - End	Observed	Mr(expt)	Mr(calc)	ppm	Miss	Sequence
6 - 17	1413.7352	1412.7279	1412.7299	-1.43	1	R.LADGDSRYYLLK.V
45 - 54	1236.6891	1235.6818	1235.6734	6.78	1	R.QRHIEDAVLR.S
47 - 54	952.5436	951.5363	951.5138	23.7	0	R.HIEDAVLR.S
55 - 78	2543.3379	2542.3306	2542.3180	4.98	0	R.SPDAIGVVIPPSQLEEAWAHIASR.Y
79 - 100	2348.1182	2347.1109	2347.1114	-0.19	0	R.YESPEALQQALDAQALDAAGMR.A
79 - 100	2364.1147	2363.1074	2363.1063	0.48	0	R.YESPEALQQALDAQALDAAGMR.A + Oxidation (M)
79 - 105	2922.4561	2921.4488	2921.4011	16.3	1	R.YESPEALQQALDAQALDAAGMRAMLAR.E + 2 Oxidation (M)
167 - 174	973.6284	972.6211	972.5968	25.1	1	R.TRIETILK.R
224 - 248	2743.4026	2742.3953	2742.3687	9.71	0	R.GELSPVLESPIGFHVLYCESVSPAR.Q
249 - 258	1211.7137	1210.7064	1210.6921	11.8	0	R.QLTLEEILPR.L

Figure S2. Determination of N-terminal sequence of TwinStrep-NifH using the standard Edman degradation method.

Blank, standard and first six amino acids were determined (mV vs retention time in mins).

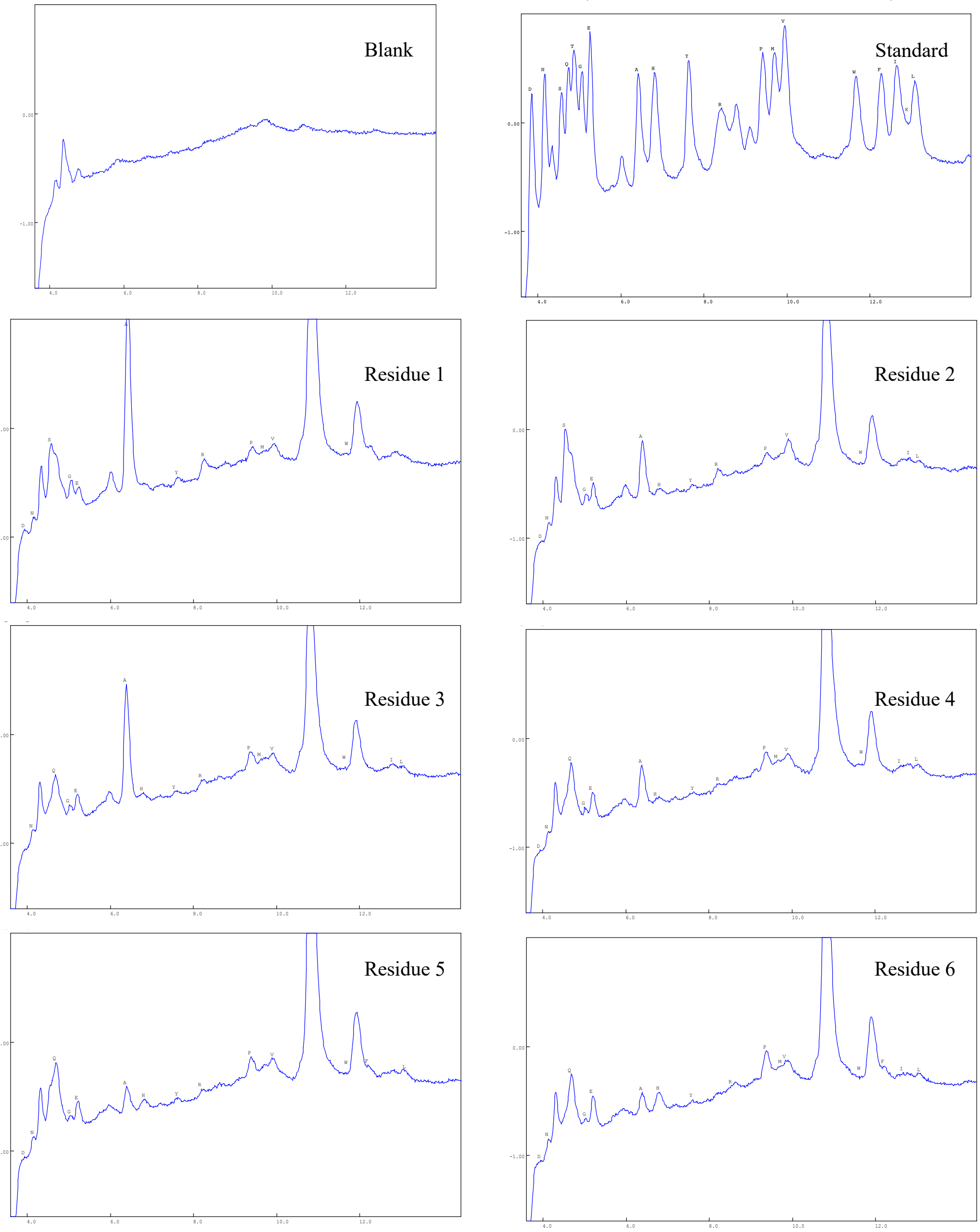

AA step	1	2	3	4	5	6
1. AA	**A**	**S**	**A**	?	**Q/S**	**H**
2. AA	S	V	P	Q	H	

Green: CTP
Red: TwinStrep
Blue: NifH
Black: cloning scars

MAAAVSTVGAINRAPLSLNGSGSGAVSAPASTFLGKKVVTVSRFAQSNKKSNGSFKVLA**ASAWSH**PQFEK
GGGSGGGSGGSAWSHPQFEKSSMAMRQCAIYGKGGIGKSTTTQNLVAALAEMGKKVMIVGCDPKADST
RLILHSKAQNTIMEMAAEAGTVEDLELEDVLKAGYGGVKCVESGGPEPGVGCAGRGVITAINFLEEEGAYED
DLDFVFYDVLGDVVCGGFAMPIRENKAQEIYIVCSGEMMAMYAANNISKGIVKYANSGSVRLGGLICNSRN
TDREDELIIALANKLGTQMIHFVPRDNVVQRAEIRRMTVIEYDPKAKQADEYRALARKVVDNKLLVIPNPIT
MDELEELLMEFGIMEVEDESIVGKTAEEV

Figure S3. NifM and NifU co-elution of with Strep-tagged NifH.

Strep-tactin purification procedure of *A. vinelandii* NifH expressed in leaves of *N. benthamiana*. CFE, cell-free extract (soluble fraction following centrifugation and filtering of disrupted leaf tissue); FT, Strep-tactin flow-through fraction; W, wash fraction; E, biotin-eluted fraction; E_c, concentrated biotin-eluted fraction. A) Western blot using antibodies against NifM, B) Western blot using antibodies against NifU, C) Western blot using antibodies against NifS. Arrows point at the correct size of the proteins, the higher mobility bands in NifS blot correspond to the signal from α-NifM wich was developed before α-NifS incubation.

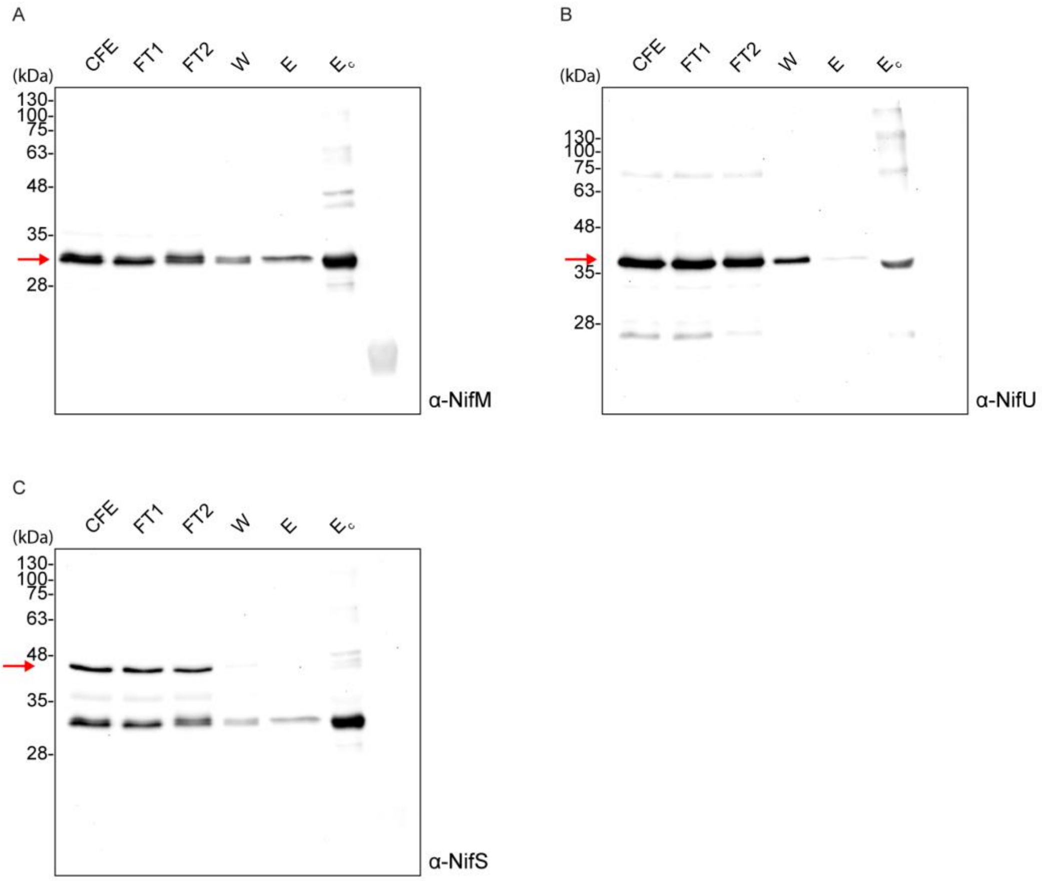

Figure S4. Confirmation of the activity of N terminal Strep tagged *A. vinelandii* NifH protein.

Strep-tactin purification procedure of TwinStrep-tagged *A. vinelandii* NifH from UW480.
CE, crude extract; CFE, cell-free extract (soluble fraction following centrifugation and filtering of lysed cells); FT, Strep-tactin flow-through fraction; W, wash fraction; E, biotin-eluted fraction.
A) Coomassie staining illustrating the purification procedure, B) Western blot using antibodies against Strep-tag, C) Western blot using antibodies against NifH, D) Determination of activities using the acetylene reduction assay, comparing the isolated TwinStrep-NifH protein with the non-tagged NifH protein (considered as 100%), both purified from *A. vinelandii*.

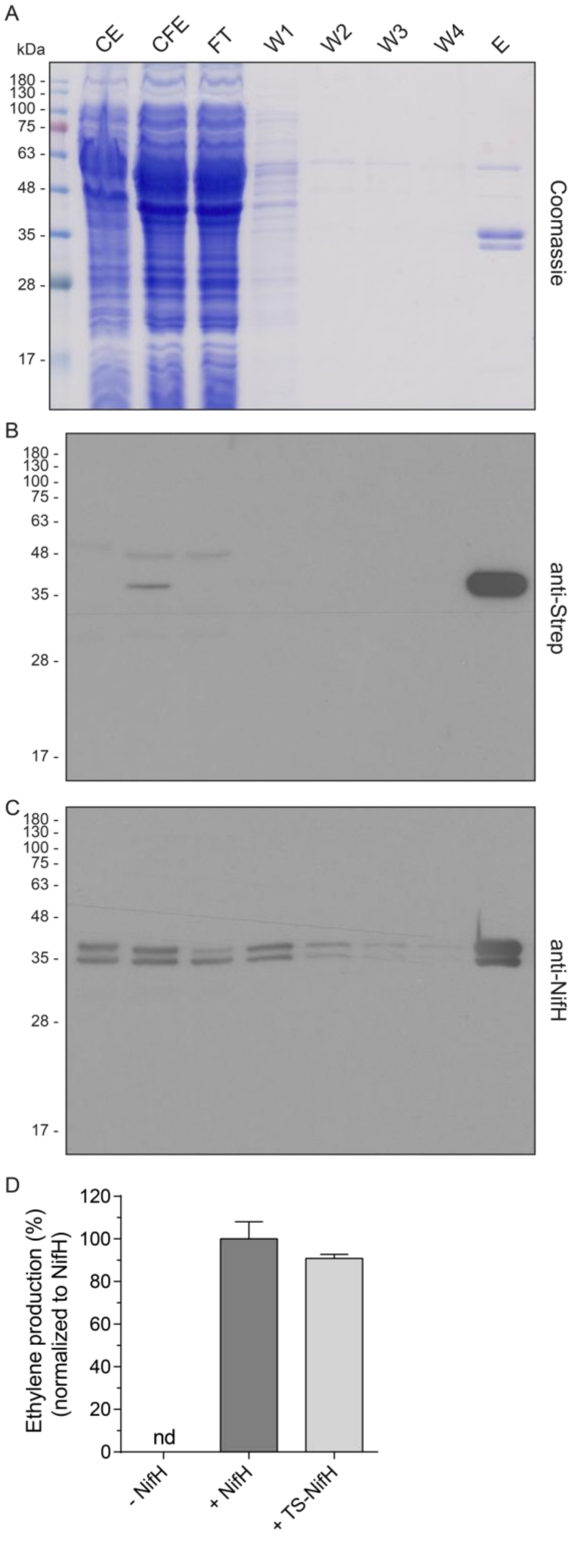

Figure S5. Purification of NifH from chloroplasts of *N. benthamiana* co-expressing *nifH* and *nifM*.

A) Schematic representation of the multigenic construct used in *N. benthamiana* transient expression assays leading to NifH purification. **B**) Coomassie staining illustrating the Strep-tactin purification procedure of *A. vinelandii* NifH expressed in leaves of *N. benthamiana*. CFE, cell-free extract (soluble fraction following centrifugation and filtering of disrupted leaf tissue); FT, Strep-tactin flow-through fraction; W, wash fraction; E, biotin-eluted fraction; Ec, concentrated eluate. **C**) Western blot analysis of the Strep-tactin purification procedure using antibodies targeting Strep-tag. Expected sizes are Twin-StrepNifH: 35.3 kDa **D**) Western blot analysis of the Strep-tactin purification procedure using antibodies targeting NifH.

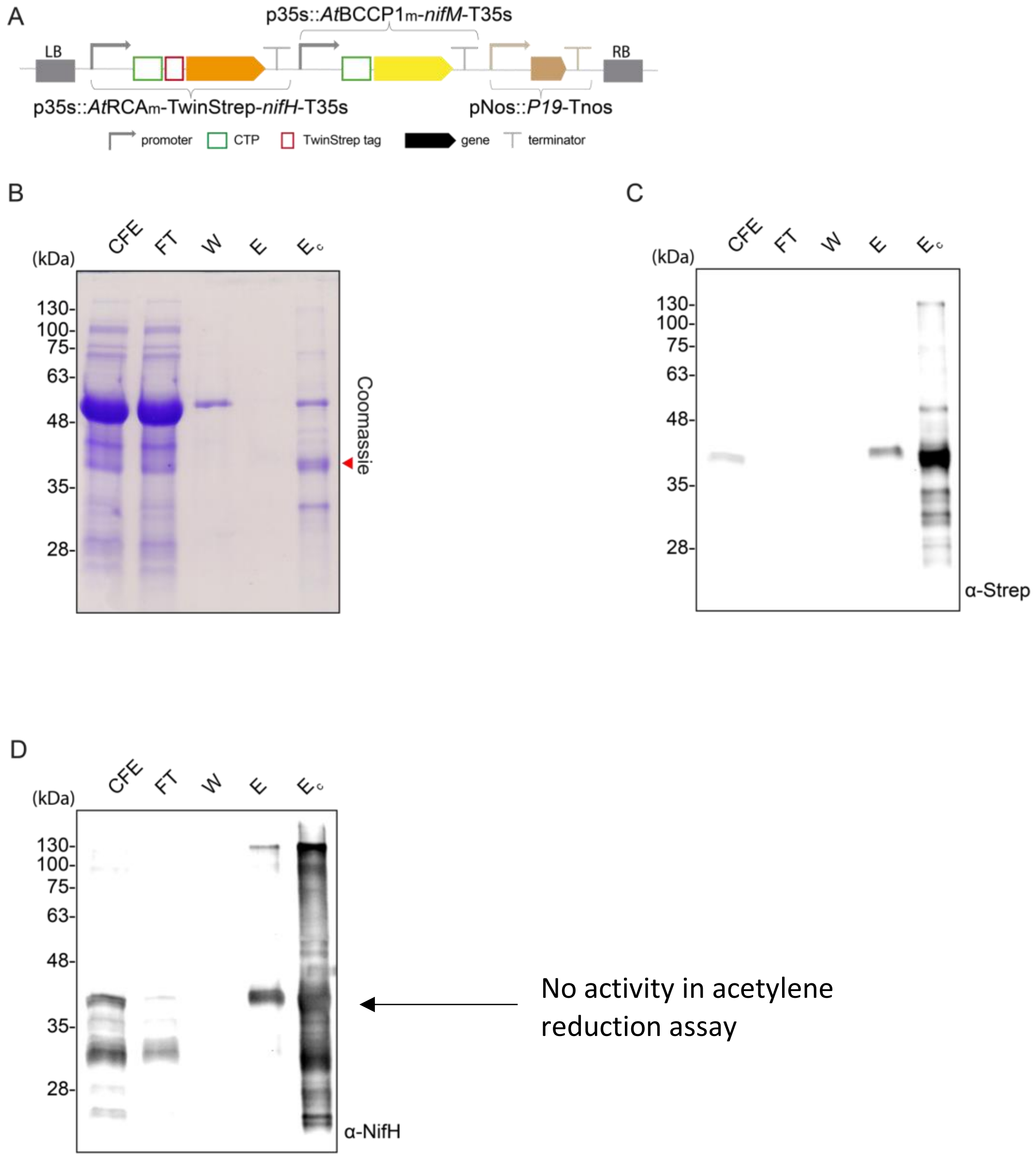

Figure S6. Mass spectrometry results of full length and truncated yeast codon optimized NifU expressed in *N. benthamiana*.

Peptide mass fingerprint Mascot search results against SwissProt database

Full length protein

Match to: **NIFU_AZOVI** Score: **101** Expect: **4.4e-005**
Nitrogen fixation protein NifU OS=Azotobacter vinelandii GN=nifU PE=3 SV=2

Nominal mass (M_r): **33764;** Calculated pI value: **4.96**

Fixed modifications: Carbamidomethyl (C)
Variable modifications: Oxidation (M)
Cleavage by Trypsin: cuts C-term side of KR unless next residue is P
Number of mass values searched: **68**
Number of mass values matched: **12**
Sequence Coverage: **51%**

Matched peptides shown in **Bold Red**

1 MWDYSEKVKE HFYNPK**NAGA VEGANAIGDV GSLSCGDALR** LTLKVDPETD
51 VILDAGFQTF GCGSAIASSS ALTEMVKGLT LDEALKISNQ DIADYLDGLP
101 PEKMHCSVMG R**EALQAAVAN YRGETIEDDH EEGALICKCF AVDEVMVR**DT
151 IRANKLSTVE DVTNYTK**AGG GCSACHEAIE RVLTEELAAR GEVFVAAPIK**
201 AKKKVK**VLAP EPAPAPVAEA PAAAPK**LSNL QRIR**RIETVL AAIRPTLQR**D
251 KGDVELIDVD GKNVYVK**LTG ACTGCQMASM TLGGIQQRLI EELGEFVK**VI
301 PVSAAAHAQM EV

Start - End	Observed	Mr(expt)	Mr(calc)	ppm	Miss	Sequence
17 - 40	2274.0339	2273.0266	2273.0706	-19	0	K.NAGAVEGANAIGDVGSLSCGDALR.L
112 - 122	1205.6151	1204.6078	1204.6200	-10	0	R.EALQAAVANYR.G
112 - 138	3002.3700	3001.3627	3001.4087	-15	1	R.EALQAAVANYRGETIEDDHEEGALICK.C
139 - 148	1225.5393	1224.5320	1224.5631	-25	0	K.CFAVDEVMVR.D
168 - 181	1474.5997	1473.5924	1473.6089	-11	0	K.AGGGCSACHEAIER.V
182 - 190	1001.5641	1000.5568	1000.5553	2	0	R.VLTEELAAR.G
182 - 200	2013.1000	2012.0927	2012.1306	-19	1	R.VLTEELAARGEVFVAAPIK.A
207 - 226	1866.9911	1865.9838	1866.0251	-22	0	K.VLAPEPAPAPVAEAPAAAPK.L
235 - 249	1737.0302	1736.0229	1736.0421	-11	1	R.RIETVLAAIRPTLQR.D
236 - 249	1580.9332	1579.9259	1579.9409	-10	0	R.IETVLAAIRPTLQR.D
268 - 288	2240.9927	2239.9854	2240.0170	-14	0	K.LTGACTGCQMASMTLGGIQQR.L
289 - 298	1176.6306	1175.6233	1175.6438	-17	0	R.LIEELGEFVK.V

Truncated protein

Match to: **NIFU_AZOVI** Score: **70** Expect: **0.055**
Nitrogen fixation protein NifU OS=Azotobacter vinelandii GN=nifU PE=3 SV=2

Nominal mass (M_r): **33764;** Calculated pI value: **4.96**
Fixed modifications: Carbamidomethyl (C)
Variable modifications: Oxidation (M)
Cleavage by Trypsin: cuts C-term side of KR unless next residue is P
Number of mass values searched: **64**
Number of mass values matched: **11**
Sequence Coverage: **32%**

Matched peptides shown in **Bold Red**

1 MWDYSEKVKE HFYNPK**NAGA VEGANAIGDV GSLSCGDALR** LTLKVDPETD
51 VILDAGFQTF GCGSAIASSS ALTEMVKGLT LDEALKISNQ DIADYLDGLP
101 PEK**MHCSVMG REALQAAVAN YRGETIEDDH EEGALICKCF AVDEVMVR**DT
151 IRANKLSTVE DVTNYTK**AGG GCSACHEAIE RVLTEELAAR GEVFVAAPIK**
201 AKKKVKVLAP EPAPAPVAEA PAAAPKLSNL QRIRRIETVL AAIRPTLQRD
251 KGDVELIDVD GKNVYVKLTG ACTGCQMASM TLGGIQQRLI EELGEFVKVI
301 PVSAAAHAQM EV

Start - End	Observed	Mr(expt)	Mr(calc)	ppm	Miss	Sequence
17 - 40	2274.0386	2273.0313	2273.0706	-17	0	K.NAGAVEGANAIGDVGSLSCGDALR.L
104 - 111	977.3661	976.3588	976.4041	-46	0	K.MHCSVMGR.E
104 - 111	993.3792	992.3719	992.3990	-27	0	K.MHCSVMGR.E Oxidation (M)
112 - 122	1205.6157	1204.6084	1204.6200	-10	0	R.EALQAAVANYR.G
112 - 138	3002.3269	3001.3196	3001.4087	-30	1	R.EALQAAVANYRGETIEDDHEEGALICK.C
139 - 148	1225.5590	1224.5517	1224.5631	-9	0	K.CFAVDEVMVR.D
139 - 148	1241.5598	1240.5525	1240.5580	-4	0	K.CFAVDEVMVR.D Oxidation (M)
168 - 181	1474.6112	1473.6039	1473.6089	-3	0	K.AGGGCSACHEAIER.V
182 - 190	1001.5667	1000.5594	1000.5553	4	0	R.VLTEELAAR.G
182 - 200	2013.0939	2012.0866	2012.1306	-22	1	R.VLTEELAARGEVFVAAPIK.A
191 - 200	1030.6079	1029.6006	1029.5859	14	0	R.GEVFVAAPIK.A

Figure S7. UV-Vis spectrum of yeast codon optimized NifU expressed in *N. benthamiana*.

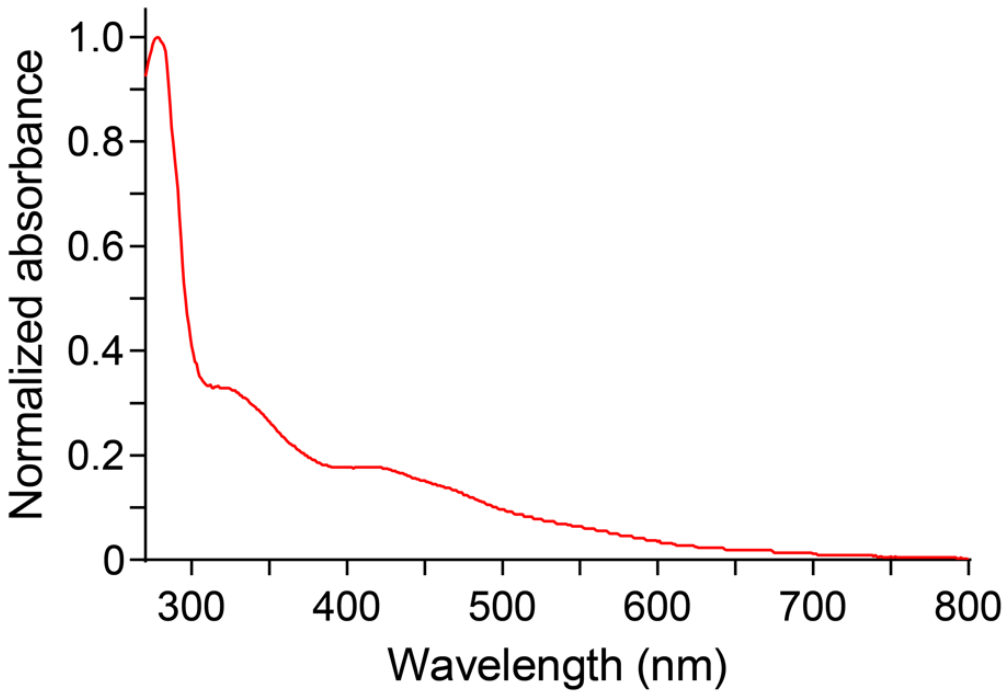

Figure S8. Expression, purification and activity assays of NifU from *N. benthamiana* chloroplasts.
A) Schematic representation of the construct used in *N. benthamiana* transient expression assays leading to NifU purification. pe35s: 35S mosaic cauliflower virus enhanced promoter; SSU: CTP from the small subunit of Rubisco; Tnos: nopaline synthase Agrobacterium terminator. **C**) Coomassie staining illustrating the Strep-tactin purification procedure of *A. vinelandii* NifU expressed in leaves of *N. benthamiana*. CE, crude extract; CFE, cell-free extract (soluble fraction following centrifugation and filtering of disrupted leaf tissue); FT, Strep-tactin flow-through fraction; W, wash fraction; E, biotin-eluted fraction. **D**) Western blot analysis of the Strep-tactin purification procedure. Antibodies targeting Strep-tag were used for visualization of the purified NifU protein.

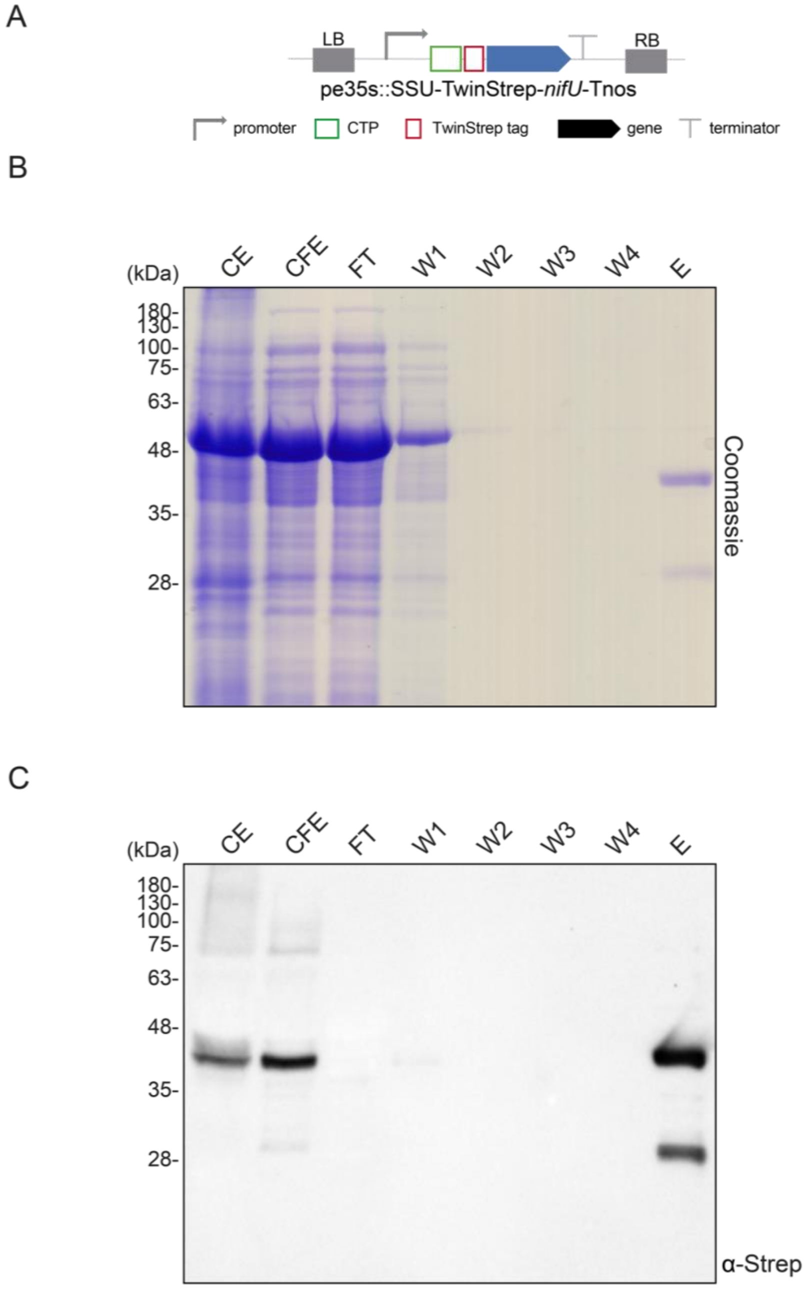

Supplement: Supplementary file 1 — Figure S1 Mass spectrometry results of bands present in NifH purification. Figure S2 Determination of N‐terminal sequence of Twin‐Strep‐NifH using the standard Edman degradation method. Figure S3 NifM and NifU co‐elution of with Strep‐tagged NifH. Figure S4 Confirmation of the activity of N‐terminal Strep‐tagged A. vinelandii NifH protein. Figure S5 Purification of NifH from chloroplasts of N. benthamiana co‐expressing nifH and nifM. Figure S6 Mass spectrometry results of full length and truncated yeast codon‐optimized NifU expressed in N. benthamiana. Figure S7 UV‐Vis spectrum of yeast codon‐optimized NifU expressed in N. benthamiana. Figure S8 Purification of NifU from chloroplasts of N. benthamiana. [file PBI-18-1882-s002.pdf]
