# Supplementary material for: Use of synthetic biology tools to optimize the production of active nitrogenase Fe protein in chloroplasts of tobacco leaf cells
Source: Plant Biotechnol J. 2020 Apr 7;18(9):1882–96. doi: 10.1111/pbi.13347 (PMC7415783; doi:10.1111/pbi.13347)
Supplement: Supplementary file 2 — Table S1 Optimization and motif settings used in Codon Optimization OnLine for nif synthetic designed genes. Table S2 Summary of Chloroplast Transit Peptides studied in this work. [file PBI-18-1882-s003.pdf]

Table S1. Optimization and motif settings used in Codon Optimization On-Line for *nif* synthetic designed genes.

Optimization settings

**Parameter**	**Setting**
Individual Codon Usage	Maximize
Codon Context	Ignore
Codon Adaptation Index	Ignore
Number of Hidden Stop Codons	Ignore
5' RNA Folding Instability	Maximize in the first 50 bp
GC Total Content	43.38%
GC3 Content	39.53%
Custom Codon Usage Pattern Values	Imported from http://www.kazusa.or.jp/codon/cgi-bin/showcodon.cgi?species=4097

Motif settings

**Parameter**	**Setting**			
Exclusion sequences	Constraint	Description	Sequence	Reference
		PolyA.1	AATGAA	Ji et al. 2007
		PolyA.2	AATATT	
		PolyA.3	GATAAA	
		PolyA.4	AATTAA	
		PolyA.5	AATAAT	
		FUE1	AATGTA	Sheng et al. 2008
		FUE2	TAGTAG	
		FUE3	TGATTC	
		FUE4	CAAGTT	
		FUE5	TAATAA	
		FUE6	TTCTTC	
		NUE1	AATAAA	
		Cryptic splice donor	MAGGTRAGT	Jackson et al. 2014
		Cryptic splice acceptor	YYYYNTAGG	
		RNA destabilizing sequence	ATTTA	
		RNA instability determinant	ATAGAT	
		Shine Dalgarno	AGGAGGTNNNNNDTG	
		ATRICH1	ASAAAA	
		ATRICH2	AASAAA	
		ATRICH3	AAASAA	
		ATRICH4	AAAASA	
		ATRICH5	TSAAAA	
		ATRICH6	TTSTTT	
		ATRICH7	TTTSTT	
		ATRICH8	TTTTST	
		NUE2	AAAAAA	
		NUE2	TTTTTT	
		FUE-TRE-CE	TTTT	

		FUE-ORY1	AAAAGT
		FUE-ORY1	TTTTGT
		FUE-ORY2	GTGTG
		FUE-ORY3	TGTAW
		FUE-ORY4	TGTGT
**Parameter**	**Setting**		
Restriction sites	Constraint	Description	Sequence
		BsaI	GGTCTC
			GAGACC
		BpiI (BbsI)	GAAGAC
			GTCTTC
		BsmBI	CGTCTC
			GCAGAG
		BtgZI	GCGATG
			CGCTAC
**Parameter**	**Setting**		
Motif Repeated Consecutively	Constraint	Description	Value
		Length of Nucleotide Motif	1
		Minimum Number of Instances before removal	12
Motif repeated Regardless of Location	Constraint	Description	Value
		Length of Nucleotide Motif	7
		Minimum Number of Instances before removal	2

Abbreviation: ICU: Individual Codon Usage; GC: Distribution of Guanine and Cytosine (GC) content in ORFs; GC3: Distribution of Guanine and Cytosine (GC) content in the third codon position; FUE: Far Upstream Element; NUE: Near Upstream Element.

Table S2. Summary of Chloroplast Transit Peptides studied in this work.

CTP name		Length	Sequence	~Mw (kDa)	Related protein (UniProt ID)	Reference
*At*AROACTP	m	1 – 42	MAQVSRICNGVQNPSLISNLSKSSQRKSPLSVSLKTQQHPRA	4.57	P05466	Klee et al., 1987
	c	1 – 76	MAQVSRICNGVQNPSLISNLSKSSQRKSPLSVSLKTQQHPRAYPISSSWGLKKSGMTLIGSELRPLKVMSSVSTAE	8.20		
*At*BCCP1CTP	m	1 – 62	MASSSFSVTSPAAAASVYAVTQTSSHFPIQNRSRRVSFRLSAKPKLRFLSKPSRSSYPVVKA	6.73	Q42533	Lee et al., 2008
	c	1 – 80	MASSSFSVTSPAAAASVYAVTQTSSHFPIQNRSRRVSFRLSAKPKLRFLSKPSRSSYPVVKAQSNKVSTGASSNAAKVDG	8.43		
*At*CAB6CTP	m	1 – 56	MASNSLMSCGIAAVYPSLLSSSKSKFVSAGVPLPNAGNVGRIRMAAHWMPGEPRPA	5.77	Q9SAG8	Lee et al., 2008<br>Lee et al., 2011
	c	1 – 67	MASNSLMSCGIAAVYPSLLSSSKSKFVSAGVPLPNAGNVGRIRMAAHWMPGEPRPAYLDGSAPGDFG	6.85		
*At*DNAJ8CTP	m	1 – 47	MTIALTIGGNGFSGLPGSSFSSSSSSFRLKNSRRKNTKMLNRSKVVC	5.01	Q9SAG8	Lee et al., 2008
	c	1 – 80	MTIALTIGGNGFSGLPGSSFSSSSSSFRLKNSRRKNTKMLNRSKVVCSSSSSVMDPYKTLKIRPDSSEYEVKKAFRQLAK	8.77		
*At*GLTB2CTP	m	1 – 73	MALQSPGATGASSSVSRLLSSAKLSSTKTIFSVDFVRSYCISKGTKRRNELSGFRGYSPLLKSSLRSPFSVKA	7.79	Q9T0P4	Lee et al., 2008
	c	1 – 80	MALQSPGATGASSSVSRLLSSAKLSSTKTIFSVDFVRSYCISKGTKRRNELSGFRGYSPLLKSSLRSPFSVKAILNSDRA	8.56		
*Nt*RBSCTP	m	1 – 20	MASSVLSSAAVATRSNVAQA	1.92	P69249	Mazur et al., 1985
	c	1 – 57	MASSVLSSAAVATRSNVAQANMVAPFTGLKSAASFPVSRKQNLDITSIASNGGRVQC	5.77		
*At*RBS1ACTP	m	1 – 55	MASSMLSSATMVASPAQATMVAPFNGLKSSAAFPATRKANNDITSITSNGGRVNC	5.53	P10795	Lee et al., 2011
	c	1 – 79	MASSMLSSATMVASPAQATMVAPFNGLKSSAAFPATRKANNDITSITSNGGRVNCMQVWPPIGKKKFETLSYLPDLTDS	8.31		
*Ps*RBS2CTP	m	1 – 22	MASMISSSAVTTVSRASRGQSA	2.18	P00869	Coruzzi et al., 1984
	c	1 – 57	MASMISSSAVTTVSRASRGQSAAVAPFGGLKSMTGFPVKKVNTDITSITSNGGRVKC	5.75		

*At*RCA$_{CTP}$	m	1 – 59	MAAAVSTVGAINRAPLSLNGSGSGAVSAPASTFLGKK<br>VVTVSRFAQSNKKSNGSFKVLA	5.81	P10896	Kim et al., 2010
	c	1 – 80	MAAAVSTVGAINRAPLSLNGSGSGAVSAPASTFLGKK<br>VVTVSRFAQSNKKSNGSFKVLAVKEDKQTDGDRWRGL<br>AYDTSD	8.25		
*Nt*SIR$_{CTP}$	m	1 – 59	MTTSFGAAINIAVADDPNPKLQIHNFSGLKSTSNSLL<br>LSRRLHVFQSFSPSNPSSIVRA	6.34	O82802	Yonekura-Sakakibara et al., 1998
	c	1 – 62	MTTSFGAAINIAVADDPNPKLQIHNFSGLKSTSNSLL<br>LSRRLHVFQSFSPSNPSSIVRAVST	6.63		
*At*TOCC$_{CTP}$	m	1 – 48	MEIRSLIVSMNPNLSSFELSRPVSPLTRSLVPFRSTK<br>LVPRSISRVSA	5.36	Q94FY7	Lee et al., 2008
	c	1 – 80	MEIRSLIVSMNPNLSSFELSRPVSPLTRSLVPFRSTK<br>LVPRSISRVSASISTPNSETDKISVKPVYVPTSPNRE<br>LRTPHS	8.88		
*syn*RBS$_{CTP}$	m	1 – 19	MASSMLSSAAVVATRASAA	1.78		Engler et al., 2014
	c	1 – 59	MASSMLSSAAVVATRASAAQASMVAPFTGLKSAASFP<br>VTRKQNNLDITSIASNGGRVRA	5.93		
*Np*SSU$_{CTP}$	c	1-60	MASSVLSSAAVATRSNVAQANMVAPFTGLKSAASFPV<br>SRKQNLDITSIASNGGRVQCMQV	6.12	P26573	Boutry et al., 2004

Abbreviation: m: minimal; c: complete.
