# Supplementary material for: Use of synthetic biology tools to optimize the production of active nitrogenase Fe protein in chloroplasts of tobacco leaf cells
Source: Plant Biotechnol J. 2020 Apr 7;18(9):1882–96. doi: 10.1111/pbi.13347 (PMC7415783; doi:10.1111/pbi.13347)
Supplement: Supplementary file 3 — Appendix S1 List of modular pieces and primers used. Appendix S2 List of assembled Transcriptional Units. Appendix S3 List of multigenic constructions. Appendix S4 Experimental Procedures from the data shown in Supporting Figures. [file PBI-18-1882-s001.pdf]

Methods S1. List of modular pieces and primers used.

**Modular piece (Level 0)**	**Amplification Primers (5’ – 3’)** (Mutagenesis primers)	**Template**
*At*mAROACTP	5’ – ATGAAGACATCTCGGGCTGCTCGTGGATGCTGCTGCGTT – 3’<br>5’ – ATGAAGACATCTCAAATGGCGCAAGTTAGCAGAATC – 3’<br>5’ – (GCGCGAAGACTGAAAACGCAGCAGCATCCACG) – 3’<br>5’ – (GCGCGAAGACCGTTTTCAGAGAAACCGATAAGGG) – 3’	Arabidopsis genomic DNA
*At*cAROACTP	5’ – ATGAAGACATCTCGGGCTGCTCGTGGATGCTGCTGCGTT – 3’<br>5’ – (ATGAAGACATCTCGGGCTTCCGCCGTGGAAACAGACGACATGACC) – 3’	Arabidopsis genomic DNA
*At*mBCCP1CTP	5’ – ATGAAGACATCTCGGGCTGCTTTCACCACAGGGTAGC – 3’<br>5’ – (ATGAAGACATCTCAAATGGCGTCGTCGTCGTTCTC) – 3’<br>5’ – (GCGCGAAGACGTCTTTCTGCTAAGCCCAAGCT) – 3’<br>5’ – (GCGCGAAGACAGAAAGACGGAAAGAAACTCTGC) – 3’	Arabidopsis cDNA
*At*cBCCP1CTP	5’ – ATGAAGACATCTCGGGCTCCATCAACTTTGGCAGCAT – 3’<br>5’ – (ATGAAGACATCTCAAATGGCGTCGTCGTCGTTCTC) – 3’<br>5’ – (GCGCGAAGACGTCTTTCTGCTAAGCCCAAGCT) – 3’<br>5’ – (GCGCGAAGACAGAAAGACGGAAAGAAACTCTGC) – 3’	Arabidopsis cDNA
*At*mDNAJ8CTP	5’ – ATGAAGACATCTCGGGCTGAACAAACGACTTTTGATCTGT – 3’<br>5’ – ATGAAGACATCTCAAATGACAATTGCTTTAACGATCGG – 3’	Arabidopsis genomic DNA
*At*cDNAJ8CTP	5’ – ATGAAGACATCTCGGGCTTTAGCGAGTTGTCTGAAAGCT – 3’<br>5’ – ATGAAGACATCTCAAATGACAATTGCTTTAACGATCGG – 3’	Arabidopsis genomic DNA
*At*mCAB6CTP	5’ – ATGAAGACATCTCGGGCTGCTGGTCGTGGCTCGCCAG – 3’<br>5’ – ATGAAGACATCTCAAATGGCGTCGAACTCGCTTATG – 3’	Arabidopsis genomic DNA
*At*cCAB6CTP	5’ – ATGAAGACATCTCGGGCTCCAAAGTCACCAGGAGCAG – 3’<br>5’ – ATGAAGACATCTCAAATGGCGTCGAACTCGCTTATG – 3’	Arabidopsis genomic DNA
*At*mGLTB2CTP	5’ – ATGAAGACATCTCGGGCTGCTTTCACCGAAAACGGCGAC – 3’<br>5’ – ATGAAGACATCTCAAATGGCTCTACAGTCTCCCGGAG – 3’<br>5’ – (ATTAAGGATCGCTTTCACCGAAAACGGCGACCTCAGCGAG) – 3’	Arabidopsis genomic DNA
*At*cGLTB2CTP	5’ – ATGAAGACATCTCAAATGGCTCTACAGTCTCCCGGAG – 3’<br>5’ – ATGAAGACATCTCGGGCTGCTCGGTCAGAATTAAGGATCG – 3’<br>5’ – (ATTAAGGATCGCTTTCACCGAAAACGGCGACCTCAGCGAG) – 3’	Arabidopsis genomic DNA
*Nt*mRBSCTP	5’ – ATGAAGACATCTCGGGCTGCTTGAGCAACATTGCTGC – 3’<br>5’ – ATGAAGACATCTCAAATGGCTTCCTCAGTTCTTTCCT – 3’	*Nicotiana tabacum* genomic DNA
*Nt*cRBSCTP	5’ – ATGAAGACATCTCGGGCTGAGCATTGCACTCTTCCGCCGT – 3’<br>5’ – ATGAAGACATCTCAAATGGCTTCCTCAGTTCTTTCCT – 3’	*Nicotiana tabacum* genomic DNA
*At*mRBS1ACTP	5’ – ATGAAGACATCTCGGGCTGAGCAGTTAACTCTTCCGCCGT – 3’<br>5’ – ATGAAGACATCTCAAATGGCTTCCTCTATGCTCTCT – 3’	Arabidopsis cDNA

*At*cRBS1A$_{CTP}$	5’ – ATGAAGACATCTCGGGCTGAATCGGTAAGGTCAGGAAGG – 3’<br>5’ – ATGAAGACATCTCAAATGGCTTCCTCTATGCTCTCT – 3’	Arabidopsis cDNA
*Ps*mRBS2$_{CTP}$	5’ – ATGAAGACATCTCGGGCTGCGGATTGCCCCCTAGAGG – 3’<br>5’ – ATGAAGACATCTCAAATGGCTTCTATGATATCCTCTTCC – 3’	*Pisum sativum* genomic DNA
*Ps*cRBS2$_{CTP}$	5’ – ATGAAGACATCTCGGGCTGAGCACTTTACTCTTCCACCAT – 3’<br>5’ – ATGAAGACATCTCAAATGGCTTCTATGATATCCTCTTCC – 3’	*Pisum sativum* genomic DNA
*At*mRCA$_{CTP}$	5’ – ATGAAGACATCTCGGGCTGCCAACACCTTGAATGATCC – 3’<br>5’ – ATGAAGACATCTCAAATGGCCGCCGCAGTTTCCAC – 3’<br>5’ – (GCGCGAAGACAAGATAAACAAACCGATGGAGAC) – 3’<br>5’ – (GCGCGAAGACTTTATCTTCTTTCACAGCCAACAC) – 3’	Arabidopsis cDNA
*At*cRCA$_{CTP}$	5’ – ATGAAGACATCTCGGGCTGAATCAGAAGTGTCGTAGGCAA – 3’<br>5’ – ATGAAGACATCTCAAATGGCCGCCGCAGTTTCCAC – 3’<br>5’ – (GCGCGAAGACAAGATAAACAAACCGATGGAGAC) – 3’<br>5’ – (GCGCGAAGACTTTATCTTCTTTCACAGCCAACAC) – 3’	Arabidopsis cDNA
*Nt*mSIR$_{CTP}$	5’ – ATGAAGACATCTCGGGCTGCGCGGACAATAGAACTAGG – 3’<br>5’ – ATGAAGACATCTCAAATGACGACGTCGTTTGGAGC – 3’<br>5’ – (GCGCGAAGACGTCTGCACGTTTTTCAGTCCTTT) – 3’<br>5’ – (GCGCGAAGACGTGCAGACGCCTGCTAAGCAAC) – 3’	*Nicotiana tabacum* genomic DNA
*Nt*cSIR$_{CTP}$	5’ – ATGAAGACATCTCGGGCTGACGTAGATACGGCGCGGACAA – 3’<br>5’ – ATGAAGACATCTCAAATGACGACGTCGTTTGGAGC – 3’<br>5’ – (GCGCGAAGACGTCTGCACGTTTTTCAGTCCTTT) – 3’<br>5’ – (GCGCGAAGACGTGCAGACGCCTGCTAAGCAAC) – 3’	*Nicotiana tabacum* genomic DNA
*At*mTOCC$_{CTP}$	5’ – ATGAAGACATCTCGGGCTGCCGAAACCCTAGAAATGG – 3’<br>5’ – ATGAAGACATCTCAAATGGAGATACGGAGCTTGATTG – 3’	Arabidopsis genomic DNA
*At*cTOCC$_{CTP}$	5’ – ATGAAGACATCTCGGGCTGAGTGAGGAGTCCGGAGTT – 3’<br>5’ – ATGAAGACATCTCAAATGGAGATACGGAGCTTGATTG – 3’	Arabidopsis genomic DNA
*syn*mRBS$_{CTP}$	5’ – ATGAAGACATCTCGGGCTGCAGCACTAGCACGAGTAG – 3’<br>5’ – ATGAAGACATCTCAAATGGCTTCTTCTATGCTTTCTT – 3’	plCH78133 (MoClo[1])
*syn*cRBS$_{CTP}$	5’ – ATGAAGACATCTCGGGCTGCTCGAACTCTTCCTCCGTTA – 3’<br>5’ – ATGAAGACATCTCAAATGGCTTCTTCTATGCTTTCTT – 3’	plCH78133 (MoClo[1])
nifH yco	5’ – ATGAAGACATCTCATTCGATGGCCATGAGACAATGTGC – 3’<br>5’ – ATGAAGACATCTCGAAGCTTATACTTCTTCAGCAGTTTTACCG – 3’	Synthesis by Proteogenix
nifM yco	5’ – ATGAAGACATCTCAAGCCATGGCCTCAGAAAGATTAG – 3’<br>5’ – ATGAAGACATCTCGAAGCTTAACCATGTGCTAAGTTTTC – 3’	Synthesis by GenScript
nifS yco	5’ – ATGAAGACATCTCAAGCCATGGCCGACGTTTACTTGGA – 3’<br>5’ – ATGAAGACATCTCGAAGCTCAACCATAGACAGGAGCAAAG – 3’<br>5’ – (AGACAGGAGCAAAGGCTTTACCAGGATCTTCAACAG) – 3’	Synthesis by GenScript

nifU yco	5’ – ATGAAGACATCTCAAGCCATGTGGGACTACTCTGAAAAGG – 3’<br>5’ – ATGAAGACATCTCGAAGCTCAGACTTCCATTTGGGCGTG – 3’	Synthesis by GenScript
nifH tsd	5’ – ATGAAGACATCTCATTCGATGGCAATGAGACAATGTGCTA – 3’<br>5’ – ATGAAGACATCTCGAAGCTTACACTTCCTCAGCGGTTT – 3’	Synthesis by NZYTech
nifM tsd	5’ – ATGAAGACATCTCAAGCCATGGCATCAGAAAGATTAGCTG – 3’<br>5’ – ATGAAGACATCTCGAAGCTTATCCATGCGCCAAATTCTCT – 3’	Synthesis by NZYTech
nifS tsd	5’ – ATGAAGACATCTCAAGCCATGGCAGACGTTTATTTGGACA – 3’<br>5’ – ATGAAGACATCTCGAAGCTTATCCGTAAACGGGCGCGAA – 3’	Synthesis by NZYTech
nifU tsd	5’ – ATGAAGACATCTCAAGCCATGTGGGATTACTCAGAGAAAG – 3’<br>5’ – ATGAAGACATCTCGAAGCTTATACTTCCATCTGAGCATG – 3’	Synthesis by NZYTech
Twin-Strep	5’ – ATGAAGACATCTCAAGCCTCTGCATGGAGTCATCCTCAG – 3’<br>5’ – ATGAAGACATCTCGCGAAGATTTTTCAAATTGTGGATGTG – 3’	Synthesis by NZYTech
p35S (1,3kb)	5’ – ATGAAGACATCTCAGGAGGAATTCCAATCCCACA – 3’<br>5’ – ATGAAGACATCTCGCATTGCGTGTCCTCTCCAAATGA – 3’	pICH51266 (MoClo[1])
p35S (1,3kb)	5’ – ATGAAGACATCTCAGGAGGAATTCCAATCCCACA – 3’<br>5’ – ATGAAGACATCTCGGGCTGCGTGTCCTCTCCAAATGA – 3’	pICH51266 (MoClo[1])
nifH tsd	5’ – ATGAAGACATCTCATTCGATGGCAATGAGACAATGTGCTA – 3’<br>5’ – ATGAAGACATCTCGCTGCCCACCGCTTCCACCACCT – 3’<br>5’ – CTTCCACCACCTGAACCTCCCACTTCCTCAGCGGTTTTGC – 3’	Synthesis by NZYTech
nifM tsd	5’ – ATGAAGACATCTCAAGCCATGGCATCAGAAAGATTAGCTG – 3’<br>5’ – ATGAAGACATCTCGCTGCCCACCGCTTCCACCACCT – 3’<br>5’ – CTTCCACCACCTGAACCTCCTCCATGCGCCAAATTCTCTA – 3’	Synthesis by NZYTech
nifS tsd	5’ – ATGAAGACATCTCAAGCCATGGCAGACGTTTATTTGGACA – 3’<br>5’ – ATGAAGACATCTCGCTGCCCACCGCTTCCACCACCT – 3’<br>5’ – CTTCCACCACCTGAACCTCCTCCGTAAACGGGCGCG – 3’	Synthesis by NZYTech
nifU tsd	5’ – ATGAAGACATCTCAAGCCATGTGGGATTACTCAGAGAAAG – 3’<br>5’ – ATGAAGACATCTCGCTGCCCACCGCTTCCACCACCT – 3’<br>5’ – CTTCCACCACCTGAACCTCCTACTTCCATCTGAGCATGTG – 3’	Synthesis by NZYTech
tGFP	5’ – ATGAAGACATCTCAGCAGATGAGAGGATCTGGATCTG – 3’<br>5’ – ATGAAGACATCTCGAAGCCTATTCCTCACCAGCATCAGC – 3’	pICSL80005 (MoClo[1])
mCherry	5’ – ATGAAGACATCTCAGCAGATGGTGAGCAAGGGC – 3’<br>5’ – ATGAAGACATCTCGAAGCTCACTTGTACAGCTCGTCCA – 3’	pICSL80007 (MoClo[1])
BFP	5’ – ATGAAGACATCTCAGCAGATGAGCGAGCTGATTAAGGAGA – 3’<br>5’ – ATGAAGACATCTCGAAGCTCAATTAAGCTTGTGCCCCAG – 3’	GB0025[2]
eGFP	-	pICH41531 (MoClo[1])
T35s	-	pICH41414 (MoClo[1])

pNOS+Ω	-	pICH87633 (MoClo[1])
p19	-	pICH44022 (MoClo[1])
tNOS	-	pICH41421 (MoClo[1])

**Not modular pieces**	**Amplification Primers (5’ – 3’)**	**Template**
pN2XJ196	(2072) – 5’ – CCACAATTTGAAAAAGGATCCTGGGACTACTCTGAAAAGG – 3’<br>(2636) – 5’ – AAAAAAGGTCACCTTAGACTTCCATTTGGGCGTGTGCG – 3’	pN2GLT4
pN2XJ197	(2423) – 5’ – AAAAAAACTAGTATGGCTTCCTCAGTTCTTTCC – 3’<br>(2430) – 5’ – CAAGTAAACGTCGGCCACCTGCATGCATTGCACTCTTC – 3’<br>(2339) – 5’ – AAAAGAGCTCTTAACCATAGACAGGAGCAAAGGCTTTACC – 3’<br>(2431) – 5’ – GCAATGCATGCAGGTGGCCGACGTTTACTTGGATAATAAC – 3’	pN2XJ196

Abbreviation: m: minimal; c: complete; yco: Yeast codon optimization; tsd: Tobacco synthetic design.
[1] Obtained from the MoClo Plant Parts Kit (Addgene: https://www.addgene.org/cloning/MoClo/patron/)
[2] Obtained from Addgene: https://www.addgene.org/68193/

Methods S2. List of assembled Transcriptional Units.

**Transcriptional Units (Level 1)**	**Entry vector**	**Modular pieces used**
*At*mAROA$_{CTP}$-*nifH*$_{tsd}$	pICH47732	p35S::*At*mAROA$_{CTP}$-Twin-Strep-*nifH*$_{tsd}$-T35s
*At*cAROA$_{CTP}$-*nifH*$_{tsd}$	pICH47732	p35S::*At*cAROA$_{CTP}$-Twin-Strep-*nifH*$_{tsd}$-T35s
*At*mBCCP1$_{CTP}$-*nifH*$_{tsd}$	pICH47732	p35S::*At*mBCCP1$_{CTP}$-Twin-Strep-*nifH*$_{tsd}$-T35s
*At*cBCCP1$_{CTP}$-*nifH*$_{tsd}$	pICH47732	p35S::*At*cBCCP1$_{CTP}$-Twin-Strep-*nifH*$_{tsd}$-T35s
*At*mDNAJ8$_{CTP}$-*nifH*$_{tsd}$	pICH47732	p35S::*At*mDNAJ8$_{CTP}$-Twin-Strep-*nifH*$_{tsd}$-T35s
*At*cDNAJ8$_{CTP}$-*nifH*$_{tsd}$	pICH47732	p35S::*At*cDNAJ8$_{CTP}$-Twin-Strep-*nifH*$_{tsd}$-T35s
*At*mCAB6$_{CTP}$-*nifH*$_{tsd}$	pICH47732	p35S::*At*mCAB6$_{CTP}$-Twin-Strep-*nifH*$_{tsd}$-T35s
*At*cCAB6$_{CTP}$-*nifH*$_{tsd}$	pICH47732	p35S::*At*cCAB6$_{CTP}$-Twin-Strep-*nifH*$_{tsd}$-T35s
*At*mGLTB2$_{CTP}$-*nifH*$_{tsd}$	pICH47732	p35S::*At*mGLTB2$_{CTP}$-Twin-Strep-*nifH*$_{tsd}$-T35s
*At*cGLTB2$_{CTP}$-*nifH*$_{tsd}$	pICH47732	p35S::*At*cGLTB2$_{CTP}$-Twin-Strep-*nifH*$_{tsd}$-T35s
*Nt*mRBS$_{CTP}$-*nifH*$_{tsd}$	pICH47732	p35S::*Nt*mRBS$_{CTP}$-Twin-Strep-*nifH*$_{tsd}$-T35s
*Nt*cRBS$_{CTP}$-*nifH*$_{tsd}$	pICH47732	p35S::*Nt*cRBS$_{CTP}$-Twin-Strep-*nifH*$_{tsd}$-T35s
*At*mRBS1A$_{CTP}$-*nifH*$_{tsd}$	pICH47732	p35S::*At*mRBS1A$_{CTP}$-Twin-Strep-*nifH*$_{tsd}$-T35s
*At*cRBS1A$_{CTP}$-*nifH*$_{tsd}$	pICH47732	p35S::*At*cRBS1A$_{CTP}$-Twin-Strep-*nifH*$_{tsd}$-T35s
*Ps*mRBS2$_{CTP}$-*nifH*$_{tsd}$	pICH47732	p35S::*Ps*mRBS2$_{CTP}$-Twin-Strep-*nifH*$_{tsd}$-T35s
*Ps*cRBS2$_{CTP}$-*nifH*$_{tsd}$	pICH47732	p35S::*Ps*cRBS2$_{CTP}$-Twin-Strep-*nifH*$_{tsd}$-T35s
*At*mRCA$_{CTP}$-*nifH*$_{tsd}$	pICH47732	p35S::*At*mRCA$_{CTP}$-Twin-Strep-*nifH*$_{tsd}$-T35s
*At*cRCA$_{CTP}$-*nifH*$_{tsd}$	pICH47732	p35S::*At*cRCA$_{CTP}$-Twin-Strep-*nifH*$_{tsd}$-T35s
*Nt*mSIR$_{CTP}$-*nifH*$_{tsd}$	pICH47732	p35S::*Nt*mSIR$_{CTP}$-Twin-Strep-*nifH*$_{tsd}$-T35s
*Nt*cSIR$_{CTP}$-*nifH*$_{tsd}$	pICH47732	p35S::*Nt*cSIR$_{CTP}$-Twin-Strep-*nifH*$_{tsd}$-T35s

*At*mTOCC$_{CTP}$-*nifH*$_{tsd}$	pICH47732	p35S::*At*mTOCC$_{CTP}$-Twin-Strep-*nifH*$_{tsd}$-T35s
*At*cTOCC$_{CTP}$-*nifH*$_{tsd}$	pICH47732	p35S::*At*cTOCC$_{CTP}$-Twin-Strep-*nifH*$_{tsd}$-T35s
*syn*mRBS$_{CTP}$-*nifH*$_{tsd}$	pICH47732	p35S::*syn*mRBS$_{CTP}$-Twin-Strep-*nifH*$_{tsd}$-T35s
*syn*cRBS$_{CTP}$-*nifH*$_{tsd}$	pICH47732	p35S::*syn*cRBS$_{CTP}$-Twin-Strep-*nifH*$_{tsd}$-T35s
*At*mAROA$_{CTP}$-*nifM*$_{tsd}$	pICH47742	p35S::*At*mAROA$_{CTP}$-*nifM*$_{tsd}$-T35s
AtcAROA$_{CTP}$-*nifM*$_{tsd}$	pICH47742	p35S::AtcAROA$_{CTP}$-*nifM*$_{tsd}$-T35s
*At*mBCCP1$_{CTP}$-*nifM*$_{tsd}$	pICH47742	p35S::*At*mBCCP1$_{CTP}$-*nifM*$_{tsd}$-T35s
*At*cBCCP1$_{CTP}$-*nifM*$_{tsd}$	pICH47742	p35S::*At*cBCCP1$_{CTP}$-*nifM*$_{tsd}$-T35s
*At*mDNAJ8$_{CTP}$-*nifM*$_{tsd}$	pICH47742	p35S::*At*mDNAJ8$_{CTP}$-*nifM*$_{tsd}$-T35s
*At*cDNAJ8$_{CTP}$-*nifM*$_{tsd}$	pICH47742	p35S::*At*cDNAJ8$_{CTP}$-*nifM*$_{tsd}$-T35s
*At*mCAB6$_{CTP}$-*nifM*$_{tsd}$	pICH47742	p35S::*At*mCAB6$_{CTP}$-*nifM*$_{tsd}$-T35s
*At*cCAB6$_{CTP}$-*nifM*$_{tsd}$	pICH47742	p35S::*At*cCAB6$_{CTP}$-*nifM*$_{tsd}$-T35s
*At*mGLTB2$_{CTP}$-*nifM*$_{tsd}$	pICH47742	p35S::*At*mGLTB2$_{CTP}$-*nifM*$_{tsd}$-T35s
*At*cGLTB2$_{CTP}$-*nifM*$_{tsd}$	pICH47742	p35S::*At*cGLTB2$_{CTP}$-*nifM*$_{tsd}$-T35s
*Nt*mRBS$_{CTP}$-*nifM*$_{tsd}$	pICH47742	p35S::*Nt*mRBS$_{CTP}$-*nifM*$_{tsd}$-T35s
*Nt*cRBS$_{CTP}$-*nifM*$_{tsd}$	pICH47742	p35S::*Nt*cRBS$_{CTP}$-*nifM*$_{tsd}$-T35s
*At*mRBS1A$_{CTP}$-*nifM*$_{tsd}$	pICH47742	p35S::*At*mRBS1A$_{CTP}$-*nifM*$_{tsd}$-T35s
*At*cRBS1A$_{CTP}$-*nifM*$_{tsd}$	pICH47742	p35S::*At*cRBS1A$_{CTP}$-*nifM*$_{tsd}$-T35s
*Ps*mRBS2$_{CTP}$-*nifM*$_{tsd}$	pICH47742	p35S::*Ps*mRBS2$_{CTP}$-*nifM*$_{tsd}$-T35s
*Ps*cRBS2$_{CTP}$-*nifM*$_{tsd}$	pICH47742	p35S::*Ps*cRBS2$_{CTP}$-*nifM*$_{tsd}$-T35s
*At*mRCA$_{CTP}$-*nifM*$_{tsd}$	pICH47742	p35S::*At*mRCA$_{CTP}$-*nifM*$_{tsd}$-T35s
*At*cRCA$_{CTP}$-*nifM*$_{tsd}$	pICH47742	p35S::*At*cRCA$_{CTP}$-*nifM*$_{tsd}$-T35s

*Nt*mSIR$_{CTP}$-*nifM*$_{tsd}$	pICH47742	p35S::*Nt*mSIR$_{CTP}$-*nifM*$_{tsd}$-T35s
*Nt*cSIR$_{CTP}$-*nifM*$_{tsd}$	pICH47742	p35S::*Nt*cSIR$_{CTP}$-*nifM*$_{tsd}$-T35s
*At*mTOCC$_{CTP}$-*nifM*$_{tsd}$	pICH47742	p35S::*At*mTOCC$_{CTP}$-*nifM*$_{tsd}$-T35s
*At*cTOCC$_{CTP}$-*nifM*$_{tsd}$	pICH47742	p35S::*At*cTOCC$_{CTP}$-*nifM*$_{tsd}$-T35s
*syn*mRBS$_{CTP}$-*nifM*$_{tsd}$	pICH47742	p35S::*syn*mRBS$_{CTP}$-*nifM*$_{tsd}$-T35s
*syn*cRBS$_{CTP}$-*nifM*$_{tsd}$	pICH47742	p35S::*syn*cRBS$_{CTP}$-*nifM*$_{tsd}$-T35s
*At*mAROA$_{CTP}$-*nifU*$_{tsd}$	pICH47761	p35S::*At*mAROA$_{CTP}$-*nifU*$_{tsd}$-T35s
AtcAROA$_{CTP}$-*nifU*$_{tsd}$	pICH47761	p35S::AtcAROA$_{CTP}$-*nifU*$_{tsd}$-T35s
*At*mBCCP1$_{CTP}$-*nifU*$_{tsd}$	pICH47761	p35S::*At*mBCCP1$_{CTP}$-*nifU*$_{tsd}$-T35s
*At*cBCCP1$_{CTP}$-*nifU*$_{tsd}$	pICH47761	p35S::*At*cBCCP1$_{CTP}$-*nifU*$_{tsd}$-T35s
*At*mDNAJ8$_{CTP}$-*nifU*$_{tsd}$	pICH47761	p35S::*At*mDNAJ8$_{CTP}$-*nifU*$_{tsd}$-T35s
*At*cDNAJ8$_{CTP}$-*nifU*$_{tsd}$	pICH47761	p35S::*At*cDNAJ8$_{CTP}$-*nifU*$_{tsd}$-T35s
*At*mCAB6$_{CTP}$-*nifU*$_{tsd}$	pICH47761	p35S::*At*mCAB6$_{CTP}$-*nifU*$_{tsd}$-T35s
*At*cCAB6$_{CTP}$-*nifU*$_{tsd}$	pICH47761	p35S::*At*cCAB6$_{CTP}$-*nifU*$_{tsd}$-T35s
*At*mGLTB2$_{CTP}$-*nifU*$_{tsd}$	pICH47761	p35S::*At*mGLTB2$_{CTP}$-*nifU*$_{tsd}$-T35s
*At*cGLTB2$_{CTP}$-*nifU*$_{tsd}$	pICH47761	p35S::*At*cGLTB2$_{CTP}$-*nifU*$_{tsd}$-T35s
*Nt*mRBS$_{CTP}$-*nifU*$_{tsd}$	pICH47761	p35S::*Nt*mRBS$_{CTP}$-*nifU*$_{tsd}$-T35s
*Nt*cRBS$_{CTP}$-*nifU*$_{tsd}$	pICH47761	p35S::*Nt*cRBS$_{CTP}$-*nifU*$_{tsd}$-T35s
*At*mRBS1A$_{CTP}$-*nifU*$_{tsd}$	pICH47761	p35S::*At*mRBS1A$_{CTP}$-*nifU*$_{tsd}$-T35s
*At*cRBS1A$_{CTP}$-*nifU*$_{tsd}$	pICH47761	p35S::*At*cRBS1A$_{CTP}$-*nifU*$_{tsd}$-T35s
*Ps*mRBS2$_{CTP}$-*nifU*$_{tsd}$	pICH47761	p35S::*Ps*mRBS2$_{CTP}$-*nifU*$_{tsd}$-T35s
*Ps*cRBS2$_{CTP}$-*nifU*$_{tsd}$	pICH47761	p35S::*Ps*cRBS2$_{CTP}$-*nifU*$_{tsd}$-T35s

*At*mRCA$_{CTP}$-*nifU*$_{tsd}$	pICH47761	p35S::*At*mRCA$_{CTP}$-*nifU*$_{tsd}$-T35s
*At*cRCA$_{CTP}$-*nifU*$_{tsd}$	pICH47761	p35S::*At*cRCA$_{CTP}$-*nifU*$_{tsd}$-T35s
*Nt*mSIR$_{CTP}$-*nifU*$_{tsd}$	pICH47761	p35S::*Nt*mSIR$_{CTP}$-*nifU*$_{tsd}$-T35s
*Nt*cSIR$_{CTP}$-*nifU*$_{tsd}$	pICH47761	p35S::*Nt*cSIR$_{CTP}$-*nifU*$_{tsd}$-T35s
*At*mTOCC$_{CTP}$-*nifU*$_{tsd}$	pICH47761	p35S::*At*mTOCC$_{CTP}$-*nifU*$_{tsd}$-T35s
*At*cTOCC$_{CTP}$-*nifU*$_{tsd}$	pICH47761	p35S::*At*cTOCC$_{CTP}$-*nifU*$_{tsd}$-T35s
*syn*mRBS$_{CTP}$-*nifU*$_{tsd}$	pICH47761	p35S::*syn*mRBS$_{CTP}$-*nifU*$_{tsd}$-T35s
*syn*cRBS$_{CTP}$-*nifU*$_{tsd}$	pICH47761	p35S::*syn*cRBS$_{CTP}$-*nifU*$_{tsd}$-T35s
*At*mAROA$_{CTP}$-*nifS*$_{tsd}$	pICH47772	p35S::*At*mAROA$_{CTP}$-*nifS*$_{tsd}$-T35s
AtcAROA$_{CTP}$-*nifS*$_{tsd}$	pICH47772	p35S::AtcAROA$_{CTP}$-*nifS*$_{tsd}$-T35s
*At*mBCCP1$_{CTP}$-*nifS*$_{tsd}$	pICH47772	p35S::*At*mBCCP1$_{CTP}$-*nifS*$_{tsd}$-T35s
*At*cBCCP1$_{CTP}$-*nifS*$_{tsd}$	pICH47772	p35S::*At*cBCCP1$_{CTP}$-*nifS*$_{tsd}$-T35s
*At*mDNAJ8$_{CTP}$-*nifS*$_{tsd}$	pICH47772	p35S::*At*mDNAJ8$_{CTP}$-*nifS*$_{tsd}$-T35s
*At*cDNAJ8$_{CTP}$-*nifS*$_{tsd}$	pICH47772	p35S::*At*cDNAJ8$_{CTP}$-*nifS*$_{tsd}$-T35s
*At*mCAB6$_{CTP}$-*nifS*$_{tsd}$	pICH47772	p35S::*At*mCAB6$_{CTP}$-*nifS*$_{tsd}$-T35s
*At*cCAB6$_{CTP}$-*nifS*$_{tsd}$	pICH47772	p35S::*At*cCAB6$_{CTP}$-*nifS*$_{tsd}$-T35s
*At*mGLTB2$_{CTP}$-*nifS*$_{tsd}$	pICH47772	p35S::*At*mGLTB2$_{CTP}$-*nifS*$_{tsd}$-T35s
*At*cGLTB2$_{CTP}$-*nifS*$_{tsd}$	pICH47772	p35S::*At*cGLTB2$_{CTP}$-*nifS*$_{tsd}$-T35s
*Nt*mRBS$_{CTP}$-*nifS*$_{tsd}$	pICH47772	p35S::*Nt*mRBS$_{CTP}$-*nifS*$_{tsd}$-T35s
*Nt*cRBS$_{CTP}$-*nifS*$_{tsd}$	pICH47772	p35S::*Nt*cRBS$_{CTP}$-*nifS*$_{tsd}$-T35s
*At*mRBS1A$_{CTP}$-*nifS*$_{tsd}$	pICH47772	p35S::*At*mRBS1A$_{CTP}$-*nifS*$_{tsd}$-T35s
*At*cRBS1A$_{CTP}$-*nifS*$_{tsd}$	pICH47772	p35S::*At*cRBS1A$_{CTP}$-*nifS*$_{tsd}$-T35s

*Ps*mRBS2$_{CTP}$-*nifS*$_{tsd}$	pICH47772	p35S::*Ps*mRBS2$_{CTP}$-*nifS*$_{tsd}$-T35s
*Ps*cRBS2$_{CTP}$-*nifS*$_{tsd}$	pICH47772	p35S::*Ps*cRBS2$_{CTP}$-*nifS*$_{tsd}$-T35s
*At*mRCA$_{CTP}$-*nifS*$_{tsd}$	pICH47772	p35S::*At*mRCA$_{CTP}$-*nifS*$_{tsd}$-T35s
*At*cRCA$_{CTP}$-*nifS*$_{tsd}$	pICH47772	p35S::*At*cRCA$_{CTP}$-*nifS*$_{tsd}$-T35s
*Nt*mSIR$_{CTP}$-*nifS*$_{tsd}$	pICH47772	p35S::*Nt*mSIR$_{CTP}$-*nifS*$_{tsd}$-T35s
*Nt*cSIR$_{CTP}$-*nifS*$_{tsd}$	pICH47772	p35S::*Nt*cSIR$_{CTP}$-*nifS*$_{tsd}$-T35s
*At*mTOCC$_{CTP}$-*nifS*$_{tsd}$	pICH47772	p35S::*At*mTOCC$_{CTP}$-*nifS*$_{tsd}$-T35s
*At*cTOCC$_{CTP}$-*nifS*$_{tsd}$	pICH47772	p35S::*At*cTOCC$_{CTP}$-*nifS*$_{tsd}$-T35s
*syn*mRBS$_{CTP}$-*nifS*$_{tsd}$	pICH47772	p35S::*syn*mRBS$_{CTP}$-*nifS*$_{tsd}$-T35s
*syn*cRBS$_{CTP}$-*nifS*$_{tsd}$	pICH47772	p35S::*syn*cRBS$_{CTP}$-*nifS*$_{tsd}$-T35s
cyto*nifH* tsd	pICH47742	p35S::Twin-Strep-*nifH*$_{tsd}$-T35s
cyto*nifM* tsd	pICH47761	p35S::*nifM*$_{tsd}$-T35s
cyto*nifS* tsd	pICH47772	p35S::*nifS*$_{tsd}$-T35s
cyto*nifU* tsd	pICH47761	p35S::*nifU*$_{tsd}$-T35s
*At*mRCA$_{CTP}$-*nifH*$_{tsd}$-*tGFP*	pICH47732	p35S::*At*mRCA$_{CTP}$-Twin-Strep-*nifH*$_{tsd}$-*tGFP*-T35s
*At*mBCCP1$_{CTP}$-*nifM*$_{tsd}$-*tGFP*	pICH47742	p35S::*At*mBCCP1$_{CTP}$-*nifM*$_{tsd}$-*tGFP*-T35s
*At*mBCCP1$_{CTP}$-*nifM*$_{tsd}$-*mCherry*	pICH47742	p35S::*At*mBCCP1$_{CTP}$-*nifM*$_{tsd}$-*mCherry*-T35s
*At*mTOCC$_{CTP}$-*nifU*$_{tsd}$-*tGFP*	pICH47761	p35S::*At*mTOCC$_{CTP}$-*nifU*$_{tsd}$-*tGFP*-T35s
*At*mCAB6$_{CTP}$-*nifS*$_{tsd}$-*tGFP*	pICH47772	p35S::*At*mCAB6$_{CTP}$-*nifS*$_{tsd}$-*tGFP*-T35s
*At*mRCA$_{CTP}$-*nifH*$_{yco}$	pICH47732	p35S::*At*mRCA$_{CTP}$-Twin-Strep-*nifH*$_{yco}$-T35s
*At*mBCCP1$_{CTP}$-*nifM*$_{yco}$	pICH47742	p35S::*At*mBCCP1$_{CTP}$-*nifM*$_{yco}$-T35s
*At*mTOCC$_{CTP}$-*nifU*$_{yco}$	pICH47761	p35S::*At*mTOCC$_{CTP}$-*nifU*$_{yco}$-T35s

*At*mCAB6$_{\text{CTP}}$-*nifS*$_{\text{yco}}$	pICH47772	p35S::*At*mCAB6$_{\text{CTP}}$-*nifS*$_{\text{yco}}$-T35s
*syn*cRBS$_{\text{CTP}}$-*BFP*	pICH47781	p35S::*syn*cRBS$_{\text{CTP}}$-*BFP*-T35s
P19	pICH47751	pNOS+Ω::*p19*-tNOS
cytoeGFP	pICH47781	p35S::*eGFP*-T35s

Abbreviation: m: minimal; c: complete; yco: Yeast codon optimization; tsd: Tobacco synthetic design.

Methods S3. List of multigenic constructions.

Multigenic constructions (Level 2)	Entry vector	Transcriptional Units used
mChloro-*nifH-nifM-nifU-nifS*-P19	pAGM4673	*At*mRCA$_{CTP}$-*nifH*$_{tsd}$ + *At*mBCCP1$_{CTP}$-*nifM*$_{tsd}$ + *At*mTOCC$_{CTP}$-*nifU*$_{tsd}$ + *At*mCAB6$_{CTP}$-*nifS*$_{tsd}$ + P19
mChloro-*nifH-nifM*-P19	pAGM4673	*At*mRCA$_{CTP}$-*nifH*$_{tsd}$ + *At*mBCCP1$_{CTP}$-*nifM*$_{tsd}$ + P19
mChloro-*nifH(tGFP)-nifM(mCherry)*-P19	pAGM4673	*At*mRCA$_{CTP}$-*nifH*$_{tsd}$-*tGFP* + *At*mBCCP1$_{CTP}$-*nifM*$_{tsd}$-*mCherry* + P19
mChloro-*nifH(tGFP)*-cChloro-*BFP*-P19	pAGM4673	*At*mRCA$_{CTP}$-*nifH*$_{tsd}$-*tGFP* + *syn*cRBS$_{CTP}$-*BFP* + P19
mChloro-*nifM(tGFP)*-cChloro-*BFP*-P19	pAGM4673	*At*mBCCP1$_{CTP}$-*nifM*$_{tsd}$-*tGFP* + *syn*cRBS$_{CTP}$-*BFP* + P19
mChloro-*nifU(tGFP)*-cChloro-*BFP*-P19	pAGM4673	*At*mTOCC$_{CTP}$-*nifU*$_{tsd}$-*tGFP* + *syn*cRBS$_{CTP}$-*BFP* + P19
mChloro-*nifS(tGFP)*-cChloro-*BFP*-P19	pAGM4673	*At*mCAB6$_{CTP}$-*nifS*$_{tsd}$-*tGFP* + *syn*cRBS$_{CTP}$-*BFP* + P19
mChloro-*nifH*$_{tsd}$-cyto*eGFP*-P19	pAGM4673	*At*mRCA$_{CTP}$-Twin-Strep-*nifH*$_{tsd}$ + cytoeGFP + P19
mChloro-*nifM*$_{tsd}$- cyto*eGFP* -P19	pAGM4673	*At*mBCCP1$_{CTP}$-*nifM*$_{tsd}$+ cytoeGFP + P19
mChloro-*nifU*$_{tsd}$- cyto*eGFP* -P19	pAGM4673	*At*mTOCC$_{CTP}$-*nifU*$_{tsd}$+ cytoeGFP + P19
mChloro-*nifS*$_{tsd}$- cyto*eGFP* -P19	pAGM4673	*At*mCAB6$_{CTP}$-*nifS*$_{tsd}$+ cytoeGFP + P19
mChloro-*nifH*$_{yco}$-cyto*eGFP*-P19	pAGM4673	*At*mRCA$_{CTP}$-Twin-Strep-*nifH*$_{yco}$+ cytoeGFP + P19
mChloro-*nifM*$_{yco}$- cyto*eGFP* -P19	pAGM4673	*At*mBCCP1$_{CTP}$-*nifM*$_{yco}$+ cytoeGFP + P19
mChloro-*nifU*$_{yco}$- cyto*eGFP* -P19	pAGM4673	*At*mTOCC$_{CTP}$-*nifU*$_{yco}$+ cytoeGFP + P19
mChloro-*nifS*$_{yco}$- cyto*eGFP* -P19	pAGM4673	*At*mCAB6$_{CTP}$-*nifS*$_{yco}$+ cytoeGFP + P19
mChloro-*nifH-nifM-nifU-nifS*- cyto*eGFP* -P19	pAGM4673	*At*mRCA$_{CTP}$-*nifH*$_{tsd}$ + *At*mBCCP1$_{CTP}$-*nifM*$_{tsd}$ + *At*mTOCC$_{CTP}$-*nifU*$_{tsd}$ + *At*mCAB6$_{CTP}$-*nifS*$_{tsd}$ + cytoeGFP + P19

Abbreviation: m: minimal; c: complete; yco: Yeast codon optimization; tsd: Tobacco synthetic design.

Methods S4. Experimental Procedures from the data shown in Supporting Figures.

Peptide mass fingerprinting. An SDS-PAGE gel with 10 µg of total protein from the concentrated NifU fraction was run and stained with Coomassie Brilliant Blue R250 (Sigma-Aldrich). Bands corresponding to full length and truncated NifU were cut and sent with ddH_2O for peptide fingerprint analysis (CAI Técnicas Biológicas, UCM, Madrid, Spain).

Edman degradation N-terminal sequencing. Three µg of pure NifH were resolved by SDS-PAGE and blotted to a PVDF membrane which was then stained with Coomassie Brilliant Blue R250 (Sigma-Aldrich) and dried. The band corresponding to NifH was cut out and sent for N-terminal sequencing (Proteome Factory AG, Berlin, Germany).

Generation and growth of a A. vinelandii strain expressing TwinStrep-tagged NifH in a ΔnifH background. Plasmid pN2XJ168 contains P*nifH* driving the expression of TwinStrep-tagged *nifH*, together with the D-sequence, all from *A. vinelandii,* and an ampicillin resistance cassette. Plasmid pN2XJ168 was generated by ELIC (Koskela and Frey, 2015), inserting the *nifH* gene into pN2SB51 in the BamHI and BstEII sites.

Transformation of pN2XJ168 into metal-starved *A. vinelandii* DJ77 strain cells (Δ*nifH*) (Jacobson et al., 1989) led to the generation of strain UW480. PCR analysis determined that, at least, two recombination events occurred: one double recombination event at P*nifH* and Δ*nifH* (rendering P*nifH*::TwinStrep-*nifH*), and one single recombination event in the D-sequence region of the *A. vinelandii genome*. The presence of TwinStrep-NifH protein and the absence of truncated NifH protein in UW480 cells was confirmed by Western blot analysis.

UW480 cells were grown in a 250 L fermentor (Bioprocess Technology S.L.) in 100L Burk's medium with urea (1.4 mM) as nitrogen source. Aeration was obtained by sparging air (120 L per minute) and stirring (110 rpm). Cells were harvested after 44 hours of growth, yielding 125 g of cells that were stored at -80ºC until used for TwinStrep-NifH isolation.

Purification and activity assay of TwinStrep-tagged A. vinelandii NifH from UW480 cells

125 g UW480 cells were resuspended in 175 ml anaerobic buffer A (100 mM Tris-HCl pH 8.3, 300 mM NaCl, 10% glycerol) supplemented with 2 mM dithionite (DTH), 1 mM PMSF, 1 µg/ml leupeptin and 5 µg/ml DNAse I. The cells were lysed in an Emulsiflex-C5 homogenizer (Avestin Inc.) operating at 20,000 lb per square inch. Cell-free extracts (CFE) were obtained by removal of cell debris and precipitated proteins by centrifugation (50,000 x g for 1 h at 4°C) and filtration

through a 0.2 µM pore size filter (Nalgene Rapid-Flow, Thermo Scientific). All procedures were performed under anaerobic conditions.

TwinStrep-NifH was purified by Strep-tag binding chromatography using a 5 ml Strep-Tactin XT Superflow Cartridge (IBA Lifesciences) under anaerobic conditions (<0.1 ppm of O2) using an AKTA Prime FPLC system (GE Healthcare) inside a glovebox (MBraun). All buffers were previously made anaerobic by sparging with N2. Before loading the cell-free extract, the Strep-Tactin column was equilibrated with buffer B (100 mM Tris-HCl pH 8.0, 300 mM NaCl, 10% glycerol, 2 mM DTH). A pH above 7.5 of the cell-free extract was ensured before loading. Cell-free extract was loaded at 2 ml/min and the column washed with four successive washes of 15 ml buffer B. Bound protein was eluted with 12 ml buffer B supplemented with 50 mM biotin, desalted using a HiPrep 26/10 Desalting column (GE Healthcare) equilibrated with buffer B, and concentrated using a 10 kDa cutoff pore centrifugal membrane device (Amicon Ultra-15, Millipore). TwinStrep-NifH protein in the elution was verified by SDS-PAGE followed by Coomassie staining and Western blotting. The purity of the isolated TwinStrep-NifH protein was estimated from Coomassie gels and quantified using ImageJ (Schneider et al., 2012). The activity of the isolated TwinStrep-NifH protein was determined using the acetylene reduction assay (Shah and Brill, 1973).

References

Jacobson, M.R., Brigle, K.E., Bennett, L.T., Setterquist, R.A., Wilson, M.S., Cash, V.L., et al. (1989) Physical and genetic map of the major nif gene cluster from Azotobacter vinelandii. J. Bacteriol., 171, 1017–1027.

Koskela, E. V. and Frey, A.D. (2015) Homologous Recombinatorial Cloning Without the Creation of Single-Stranded Ends: Exonuclease and Ligation-Independent Cloning (ELIC). Mol. Biotechnol., 57, 233–240.

Schneider, C.A., Rasband, W.S., and Eliceiri, K.W. (2012) NIH Image to ImageJ: 25 years of image analysis. Nat. Methods, 9, 671–675.

Shah, V.K. and Brill, W.J. (1973) Nitrogenase. IV. Simple method of purification to homogeneity of nitrogenase components from Azotobacter vinelandii. Biochim. Biophys. Acta, 305, 445–454.
